# Hypertonic saline mediates the NLRP3/IL‐1β signaling axis in microglia to alleviate ischemic blood‐brain barrier permeability by downregulating astrocyte‐derived VEGF in rats

**DOI:** 10.1111/cns.13427

**Published:** 2020-06-12

**Authors:** Qiao‐sheng Wang, Hong‐guang Ding, Sheng‐long Chen, Xin‐qiang Liu, Yi‐yu Deng, Wen‐qiang Jiang, Ya Li, Lin‐qiang Huang, Yong‐li Han, Miao‐yun Wen, Mei‐qiu Wang, Hong‐ke Zeng

**Affiliations:** ^1^ Department of Critical Care Medicine The First Affiliated Hospital, University of South China Hengyang China; ^2^ Department of Emergency and Critical Care Medicine Guangdong Provincial People’s Hospital, Guangdong Academy of Medical Sciences Guangzhou China; ^3^ School of Medicine South China University of Technology Guangzhou China

**Keywords:** blood‐brain barrier, hypertonic saline, microglia, NLRP3 inflammasome, stroke

## Abstract

**Introduction:**

The aim of this study was to explore whether the antibrain edema of hypertonic saline (HS) is associated with alleviating ischemic blood‐brain barrier (BBB) permeability by downregulating astrocyte‐derived vascular endothelial growth factor (VEGF), which is mediated by microglia‐derived NOD‐like receptor protein 3 (NLRP3) inflammasome.

**Methods:**

The infarct volume and BBB permeability were detected. The protein expression level of VEGF in astrocytes in a transient focal brain ischemia model of rats was evaluated after 10% HS treatment. Changes in the NLRP3 inflammasome, IL‐1β protein expression, and the interleukin‐1 receptor (IL1R1)/pNF‐кBp65/VEGF signaling pathway were determined in astrocytes.

**Results:**

HS alleviated the BBB permeability, reduced the infarct volume, and downregulated the expression of VEGF in astrocytes. HS downregulates IL‐1β expression by inhibiting the activation of the NLRP3 inflammasome in microglia and then downregulates VEGF expression by inhibiting the phosphorylation of NF‐кBp65 mediated by IL‐1β in astrocytes.

**Conclusions:**

HS alleviated the BBB permeability, reduced the infarct volume, and downregulated the expression of VEGF in astrocytes. HS downregulated IL‐1β expression via inhibiting the activation of the NLRP3 inflammasome in microglia and then downregulated VEGF expression through inhibiting the phosphorylation of NF‐кBp65 mediated by IL‐1β in astrocytes.

## Graphical abstract

Hypertonic saline inhibits activation of NLRP3 inflammasome in microglia to alleviate ischemic blood‐brain barrier permeability by downregulating astrocyte‐derived VEGF.

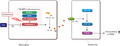

## 1 INTRODUCTION

Vascular brain edema is secondary to increased blood‐brain barrier (BBB) permeability following cerebral ischemia and can deteriorate neurological function and endanger patients’ lives.^1^, ^2^ BBB dysfunction is also central to the genesis of hemorrhagic transformation and increased mortality in stroke.^3^ Vascular endothelial growth factor (VEGF) is an important factor that increases BBB permeability. VEGF expression in astrocytes and BBB permeability can be increased by central nervous system immunological diseases, hypoxia, and cerebral ischemia.^4^, ^5^, ^6^, ^7^ Our previous study found that upregulating astrocyte‐derived VEGF increases BBB permeability in a transient focal cerebral ischemia model in rats.^8^

The neuroinflammatory response is one of the important mechanisms of nerve injury that occurs after cerebral ischemia and increased BBB permeability.^9^ Brain‐resident microglial cells are activated within the first few hours after ischemia and release pro‐inflammatory cytokines.^10^ It has been found that intracellular NOD‐like receptors are widely expressed in microglia, especially intracellular NOD‐like receptor protein 3 (NLRP3), which plays an important role in initiating the central inflammatory response.^11^, ^12^, ^13^ Activation of the NLRP3 inflammasome can upregulate the expression of interleukin‐1β (IL‐1β) and then promotes the central nervous system cascade inflammatory response leading to the aggravation of nerve injury in ischemic stroke patients.^14^, ^15^

Studies have confirmed that compared with classic mannitol, hypertonic saline (HS) has significant advantages in the treatment of cerebral edema and intracranial hypertension resulting from various causes, including cerebral ischemia.^16^, ^17^, ^18^, ^19^, ^20^ Besides the mechanism of osmotic dehydration, HS also has the effect of antibrain edema through the mechanism of non‐osmotic dehydration, such as antiinflammatory.^8^, ^21^

In recent years, it has been found that the crosstalk between microglia and astrocytes plays a central role in regulating inflammation in the brain. Activated microglia can release inflammatory factors (ie, IL‐1β), which can activate astrocytes and upregulate glial fibrillary acidic protein (GFAP) expression.^22^, ^23^, ^24^ Our previous study demonstrated that HS can inhibit microglia‐derived IL‐1β in focal brain ischemia in rats. However, the internal molecular mechanism has remained unclear. The aim of this study was to explore whether the antibrain edema of HS is associated with alleviating ischemic BBB permeability by downregulating astrocyte‐derived VEGF, which was mediated by microglia‐derived NLRP3 inflammasomes in rats.

## 2 MATERIALS AND METHODS

### 2.1 Animals and groups

Adult male Sprague Dawley (SD) rats aged 3‐4 months and weighing 250‐300 g were randomly divided into four groups (n = 32 rats each): sham‐operated group (sham group), cerebral ischemia‐reperfusion group (IR group), cerebral ischemia‐reperfusion + normal saline group (NS group), and cerebral ischemia‐reperfusion + 10% HS group (HS group). The rats were further divided into 12‐ and 24‐hour subgroups according to the reperfusion time. The rats in the sham group and IR group received no therapeutic intervention. The rats in the NS group and HS group received 0.3 mL 0.9% normal saline or 10% HS through the caudal vein at the beginning of reperfusion until the end of the experiment. All animal experimental procedures were approved by the Institutional Animal Care and Use Committee of Guangdong Province, China (No. GBREC2012106A(R1)).

### 2.2 BV‐2 microglial cell culture and groups

BV‐2 microglial cells were identified by lectin (Sigma; L0401); cells were then cultured in Dulbecco's modified Eagle's medium‐F12 nutrient mixture (DMEM‐F12) supplemented with 10% fetal bovine serum (FBS). The cells were divided into a control group, an oxygen‐glucose deprivation (OGD) group, and an OGD + HS group. The cells in the OGD group were cultured for 2 hours with glucose‐free medium in an airtight hypoxia chamber with 3% O_2_/5% CO_2_ at 37°C, and then, the cells were switched to the culture medium with 10% FBS for 24 hours. In this study, we chose 80 mmol/L HS as described.^25^, ^26^ The cells in the HS group were cultured with the 2 mL culture medium containing 10% FBS and 80 mmol/L HS for 24 hours after 2‐hour OGD. However, the cells in the control group were cultured with only the culture medium containing 10% FBS.

### 2.3 TNC1 astrocyte culture and groups

DI TNC1 cells (ATCC, CRL‐2005™) were maintained in 75‐cm^2^ culture flasks with DMEM‐F12 supplemented with 10% FBS. The cultures were incubated at 37°C in a humidified incubator under 5% CO_2_.

### 2.4 **Group** Ⅰ

To prove that HS downregulated the expression of VEGF protein, which may be associated with inhibiting the IL‐1β/IL1R1/nuclear transcription factor kappa B (NF‐κB) signaling pathway in astrocytes, astrocytes were incubated with the culture medium used to incubate the microglia of the control group, the OGD group, and the HS group as mentioned in the above experiment for 24 hours. Based on the above, the interleukin‐1 receptor antagonist (IL1Ra) + OGD group (IL1Ra group, 40 ng/mL) was added to clarify the above mechanism. After incubation with the above‐mentioned reagents for 24 hours, the cells were harvested for Western blotting and immunofluorescence microscopy (6‐well plates).

### 2.5 **Group** Ⅱ

An IL‐1β concentration of 40 ng/mL was used in the following experiment, which was determined by TNC1 astrocyte viability detected by the CCK‐8 assay (cell counting kit‐8) according to the manufacturer's instructions (Figure S5B). TNC1 astrocytes were divided into a control group, IL‐1β group (40 ng/mL; Peprotech Inc 400‐01B), IL1Ra (40 ng/mL; Peprotech Inc 200‐01RA) group, IL‐1β + IL1Ra (added for 30 minutes before addition of IL‐1β) group, IL‐1β + SN50 (18 μmol/L, an inhibitor of NF‐κB, MedChemExpress, Cat.No.213546‐53‐3, added for 30 minutes before addition of IL‐1β) group, and SN50 group based on treatment with the respective conditioned medium. TNC1 cells were seeded at a density of 3.0 × 10^5^ cells/well in 6‐well plates incubated with complete medium. After 24 hours, the cells were incubated with 2 mL (6‐well plates) of conditioned medium for 24 hours. The cells were then harvested for protein extraction and immunofluorescence microscopy. The schematic diagram depicts the design and flow chart of the cell experiment (Figure S8).

### 2.6 Transient focal brain ischemia model established by the intraluminal suture method

Transient focal brain ischemia of right middle cerebral artery occlusion (MCAO) was induced as described previously.^18^, ^27^ Briefly, after being anaesthetized, the right common carotid artery (CCA), external carotid artery (ECA), and internal carotid artery (ICA) were exposed. The CCA was temporarily blocked, and the distal end of the ECA was severed. Then, the occluder was inserted from the distal ECA and advanced into the ICA 17‐19 mm. The occluder was withdrawn to the ECA to restore MCA blood flow after 2 hours MCAO. Sham‐operated rats were subjected to the surgical procedures except for transient MCAO. Neurological deficits were evaluated by the Longa scores test.

### 2.7 Permeability of the blood‐brain barrier in the ischemic cerebral hemisphere detected by Evans blue

The rats were injected with 4 mL/kg of 2% Evans blue via the caudal vein before 1 hour at the end of the experiment. Then, the rats were transcardially perfused with normal saline and 4% polyformaldehyde. Next, 2‐mm‐thick brain coronal slices were made consecutively and dehydrated with a 20% and 30% sucrose gradient for 24 hours. Brain coronal frozen sections of 10 µm thickness were cut. After rinsing in PBS, the sections were mounted with a fluorescent mounting medium and were observed under a fluorescence microscope as previously described.^28^ The content of Evans blue in the brain tissue was measured quantitatively by an ultraviolet spectrophotometer.

### 2.8 Western blotting

Total proteins were extracted. Protein samples were separated on polyacrylamide‐SDS gels and electroblotted onto nitrocellulose membranes. After blocking with 5% non‐fat milk, the membranes were incubated overnight with the following primary antibodies: anti‐VEGF (1:500; Santa Cruz Biotechnology, sc‐152), anti‐NLRP3 (2 μg/mL; Novus Biologicals, NBP2‐12446), anti‐ASC (1:1000; Santa Cruz Biotechnology, sc‐22514‐R), anti‐caspase‐1 (1:500; Santa Cruz Biotechnology, sc‐154), anti‐pro‐IL‐1β (1:400; Proteintech Group, 16806‐1‐AP), anti‐IL‐1β (0.2 μg/mL; Abcam, ab9787), anti‐IL1R1 (1:500; Santa Cruz Biotechnology, sc‐689), anti‐NF‐кBp65 (Cell Signaling Technology, #8242), anti‐pNF‐кBp65 (1:100; Cell Signaling Technology, #3033), and anti‐β‐actin (1:1000; Cell Signaling Technology, #3700). After washing three times, they were incubated with the appropriate HRP‐conjugated secondary antibody (1:1000; Cell Signaling Technology, #7074 or #7076) for 45 minutes at room temperature. The immunoblots were developed using the enhanced chemiluminescence detection system. The band intensity was quantified by ImageJ 1.39u software.

### 2.9 Immunofluorescence

Rats under deep anesthesia were transcardially perfused with normal saline and 4% paraformaldehyde. The brains were dehydrated with 20% and 30% sucrose. Ten‐micron‐thick brain sections were cut and blocked with 10% bovine serum albumin (BSA). The sections were then incubated overnight with primary antibodies directed against VEGF (1:500; Santa Cruz Biotechnology, sc‐152), NLRP3 (1:50; Santa Cruz Biotechnology, sc‐74694), ASC (1:100; Santa Cruz Biotechnology, sc‐22514‐R), caspase‐1 (1:50; Santa Cruz Biotechnology, sc‐154), IL‐1β (1:400; Abcam. ab9787), IL1R1 (1:100; Santa Cruz Biotechnology. sc‐689), GFAP (1:50, monoclonal antibody IgG; Millipore, MAB 360), and lectin (1:100; Sigma; L0401). The next day, the sections were incubated with the secondary antibodies as follows: Alexa Fluor^®^ 488‐conjugated goat anti‐mouse IgG (1:100; Thermo Fisher Scientific; A‐28175), Alexa Fluor^®^ 555‐conjugated donkey anti‐rabbit IgG (1:100; Thermo Fisher Scientific; A‐31572), Alexa Fluor^®^ 488‐conjugated donkey anti‐rabbit IgG (1:100; Thermo Fisher Scientific Inc; A‐21206), and Alexa Fluor^®^ 555‐conjugated goat anti‐mouse IgG (1:100; Thermo Fisher Scientific; A‐21424). Then, the slides were mounted with a fluorescent mounting medium. Colocalization was observed with a fluorescence microscope. The peri‐infarct and infarct region were detected with high consistency and accuracy.

In addition, the BV‐2 cells and TNC1 cells in culture were processed for immunofluorescence labeling. The cells were fixed in 4% paraformaldehyde and then blocked with 5% BSA. Subsequently, the BV‐2 cells were incubated overnight with the following primary antibodies: anti‐NLRP3, anti‐ASC, anti‐caspase‐1, anti‐IL‐1β, and lectin. TNC1 cells were incubated with the following primary antibodies: anti‐VEGF, anti‐IL1R1, anti‐VEGF, and anti‐GFAP. The following day, the cells were incubated with the secondary antibodies: Alexa Fluor^®^ 488‐conjugated donkey anti‐rabbit IgG (1:100) and Alexa Fluor^®^ 555‐conjugated goat anti‐mouse IgG (1:100). Following rinsing in PBS, they were mounted with a fluorescent mounting medium. Colocalization was observed with a fluorescence microscope.

### 2.10 Magnetic Resonance Imaging (MRI)

MRI measurements were performed at 24 hours post‐MCAO using a 7.0T scanner (Bruker BioSpin) with a 30‐mm‐diameter rat head coil. T2‐weighted MRI was performed with the following parameters: slice thickness (THK) = 1 mm, interslice gap = 0.5 mm, field of view (FOV) = 3.0 × 3.0 cm^2^, matrix = 256 × 256, repetition time (TR) = 1000 ms, echo time (TE) = 50 ms. The infarct volume was calculated on T2WI maps using ImageJ software. DTI parameters were THK = 1 mm, interslice gap = 0.5 mm, FOV = 3.0 × 3.0 cm^2^, matrix = 128 × 128, TR = 3000 ms, TE = 32 ms, Δ = 20 ms, δ = 4 ms, in‐plane image resolution = 250 × 250 μm^2^, NEX = 4, 30 gradient directions, and *b* values = 0, 1000 s/mm^2^. To compensate for the effects of brain swelling, apparent diffusion coefficient (ADC) maps were produced. Relative ADC (rADC) was calculated using the following equation:

$$\text{Re}lative\;ADC=\frac{ADC\;of\;\inf arcted\;hemisphere}{ADC\;of\;contralateral\;hemisphere}\times 100.$$

### 2.11 IL‐1β of BV‐2 microglial cell cultures evaluation using ELISA

The IL‐1β levels were evaluated using enzyme‐linked immunosorbent assay (ELISA) kits (Abcam; Cat. No. ab197742) following the manufacturers’ instructions. Briefly, the samples and standards were added to the plate wells coated by IL‐1β antibodies labeled with HRP. After this, a TMB substrate solution was pipetted to the wells. Then, the stop buffer was added, and the optical density (OD) was measured spectrophotometrically at a wavelength of 450 nm. The concentrations of IL‐1β in the samples were determined by comparing the optical density of the samples with the standard curve.

### 2.12 Statistical analysis

SPSS 19.0 (IBM) was utilized to analyze all data. The results are expressed as the means ± standard deviations. Differences among multiple groups were statistically analyzed by one‐way ANOVA if the data were homogeneity of variance; otherwise, they were analyzed by Welch ANOVA. Multiple comparisons were analyzed by the least significant difference (LSD) method if the data were homogeneity of variance; otherwise, they were analyzed by Dunnett's T3 method. Values of *P* < .05 were considered statistically significant.

Three‐group univariate‐factor measurement data were analyzed.

## 3 RESULTS

### 3.1 The effect of HS on infarct size and brain edema

**FIGURE 1:**
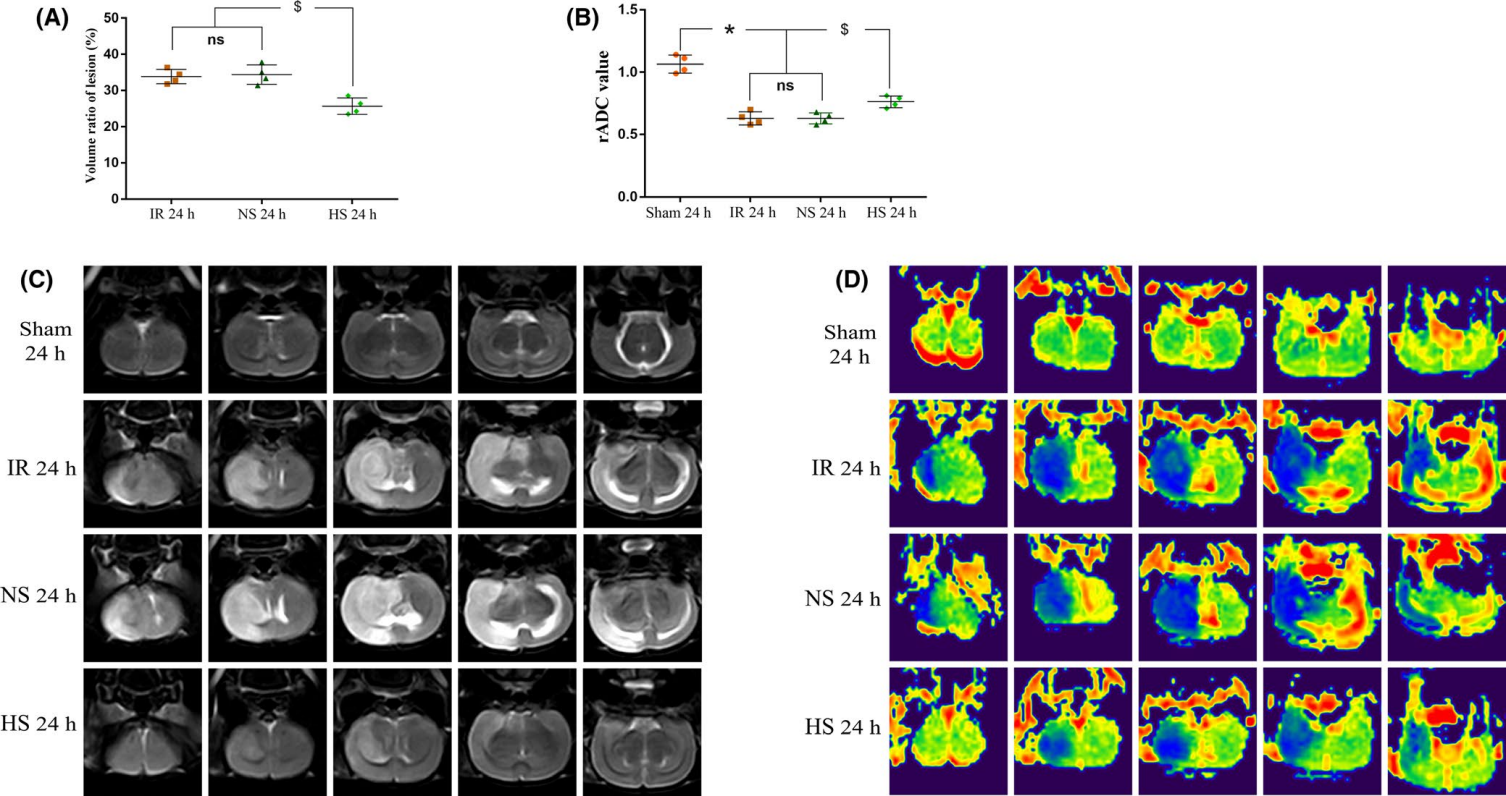
The effect of HS on the infarct size and brain edema. T2WI images (C) at 24 h after MCAO or sham surgery from NS‐treated and HS‐treated rats. A, The volume ratio of lesion was much smaller in HS group compared with IR group (^$^ *P* < .05) and NS group (^$^ *P* < .05). ADC images (D) at 24 h after MCAO or sham surgery from NS‐treated and HS‐treated rats. B, The rADC calculated by software from DTI sequence. The level of rADC was much higher in the sham group and HS group compared with IR group (^$^ *P* < .05) and NS group (^$^ *P* < .05). The values represent the means ± SD, n = 4; significant differences are expressed as *^$^ *P* < .05; ns: non‐significant, *P* > .05

T2WI images from rats in each group at 24 hours (Figure 1C). The volume ratio of lesion was much smaller in HS group compared with IR group (^$^ *P* < .05) and NS group (^$^ *P* < .05) (Figure 1A). ADC images from rats in each group at 24 hours (Figure 1D). The rADC calculated by software from DTI sequence. The level of rADC was much higher in HS group compared with IR group (^$^ *P* < .05) and NS group (^$^ *P* < .05) (Figure 1B).

### 3.2 HS alleviates blood‐brain barrier permeability in ischemic hemisphere brain tissue

**FIGURE 2:**
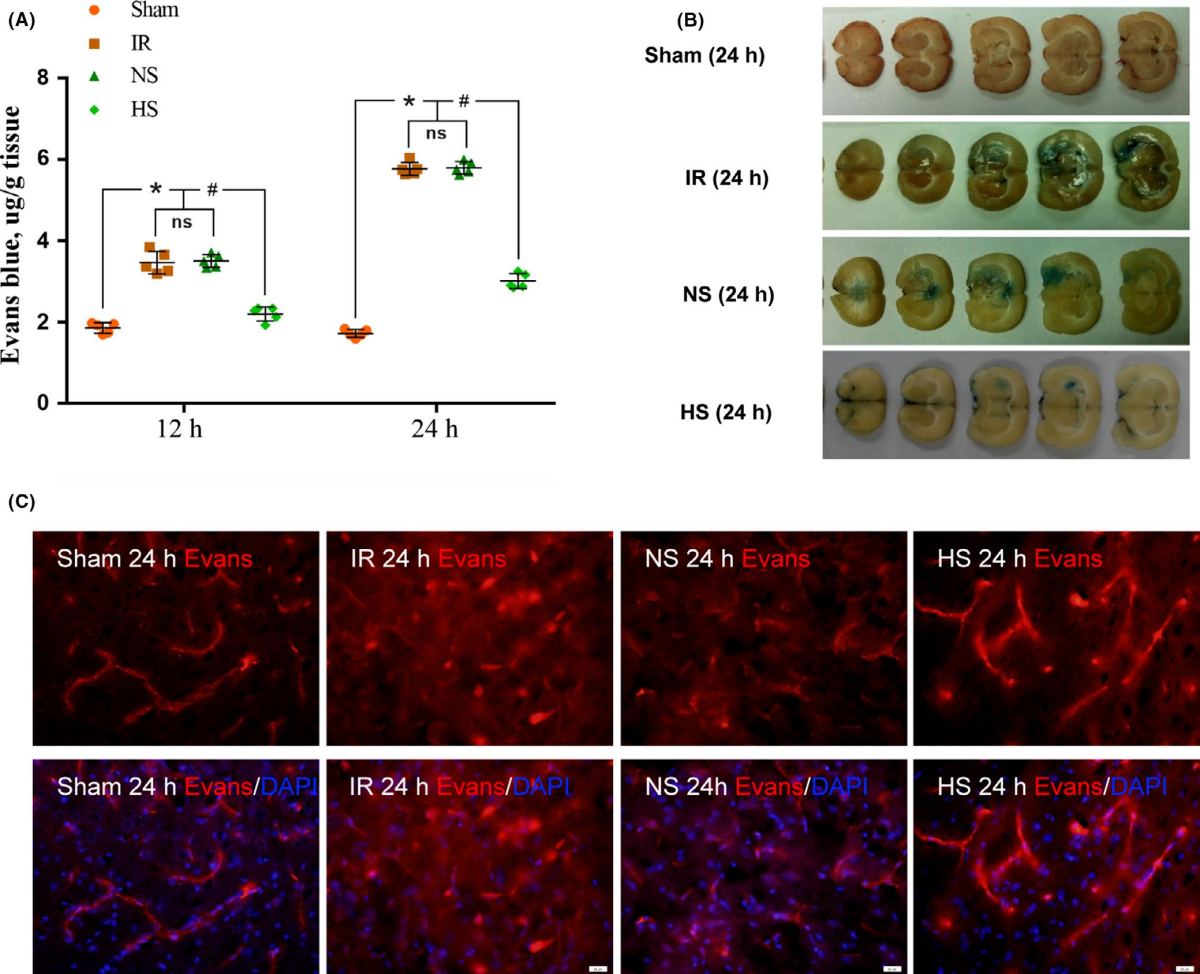
Permeability of the brain‐blood barrier reflected by Evans blue extravasation in the ischemic cerebral hemisphere among different groups. The dot plot (A) shows the content of Evans blue by quantitative analysis at 12 and 24 h in different groups. Compared with the Evans blue extravasation of the ischemic cerebral hemisphere in the IR and NS groups, Evans blue was decreased significantly at 12 and 24 h in the HS group (*P < .05 and ^#^ *P* < .05 vs the IR and NS groups). The values represent the means ± SD, n = 5; ns: non‐significant, *P* > .05. The blue area indicates the extravasation of Evans blue in the ischemic cerebral hemisphere of each group. The Evans blue extravasation of brain tissue in the ischemic cerebral hemisphere was markedly increased in the IR 24‐h group and the NS 24‐h group compared with that in the sham 24‐h group; however, it was evidently decreased in the HS 24‐h group (B). The fluorescence images in C show that the red fluorescence was confined to the cerebral microvessels of the ischemic cerebral hemisphere in the sham 24‐h group; however, massive Evans blue extravasation occurred and inundated the brain tissues in the IR 24‐h group and the NS 24‐h group. At 24 h after treatment with 10% HS, Evans blue extravasation appeared to diminish. Scale bars in panel C: 20 μm. n = 3 for each group

Figure 2A shows that the Evans blue content detected quantitatively by fluorescence spectrophotometry in the ischemic hemisphere cerebral tissue of rats in the IR and NS groups increased significantly compared with the content in the corresponding regions of the cerebral hemisphere in rats from the sham group 12 and 24 hours after reperfusion (*P < .05); however, the Evans blue content decreased significantly after HS treatment for 12 and 24 hours, respectively (^#^ *P* < .05). Coronary brain slices (Figure 2B) showed that compared with the extravasation in the IR and NS groups, the extension of blue Evans blue extravasation in the ischemic hemisphere brain tissue decreased obviously 24 hours after HS treatment. In the case of increased BBB permeability, Evans blue dye in cerebrovascular tissues can seep out into brain tissues and can be shown to be red by fluorescence microscopy.^28^, ^29^ Fluorescence imaging (Figure 2C) shows that a clear blood vessel outline (red) can be observed in the corresponding area of ischemic hemisphere brain tissue and indicates that there was no Evans blue extravasation into the peripheral brain tissue in the sham group at 24 hours. However, a large amount of red Evans blue was extravasated into ischemic hemisphere brain tissues of the IR and NS groups 24 hours after reperfusion, but the extension of red Evans blue extravasation decreased significantly 24 hours after HS treatment. This result indicates that HS can decrease the permeability of the BBB induced by ischemic stroke in rats.

### 3.3 HS downregulates the expression of VEGF in astrocytes of peri‐ischemic brain tissue

**FIGURE 3:**
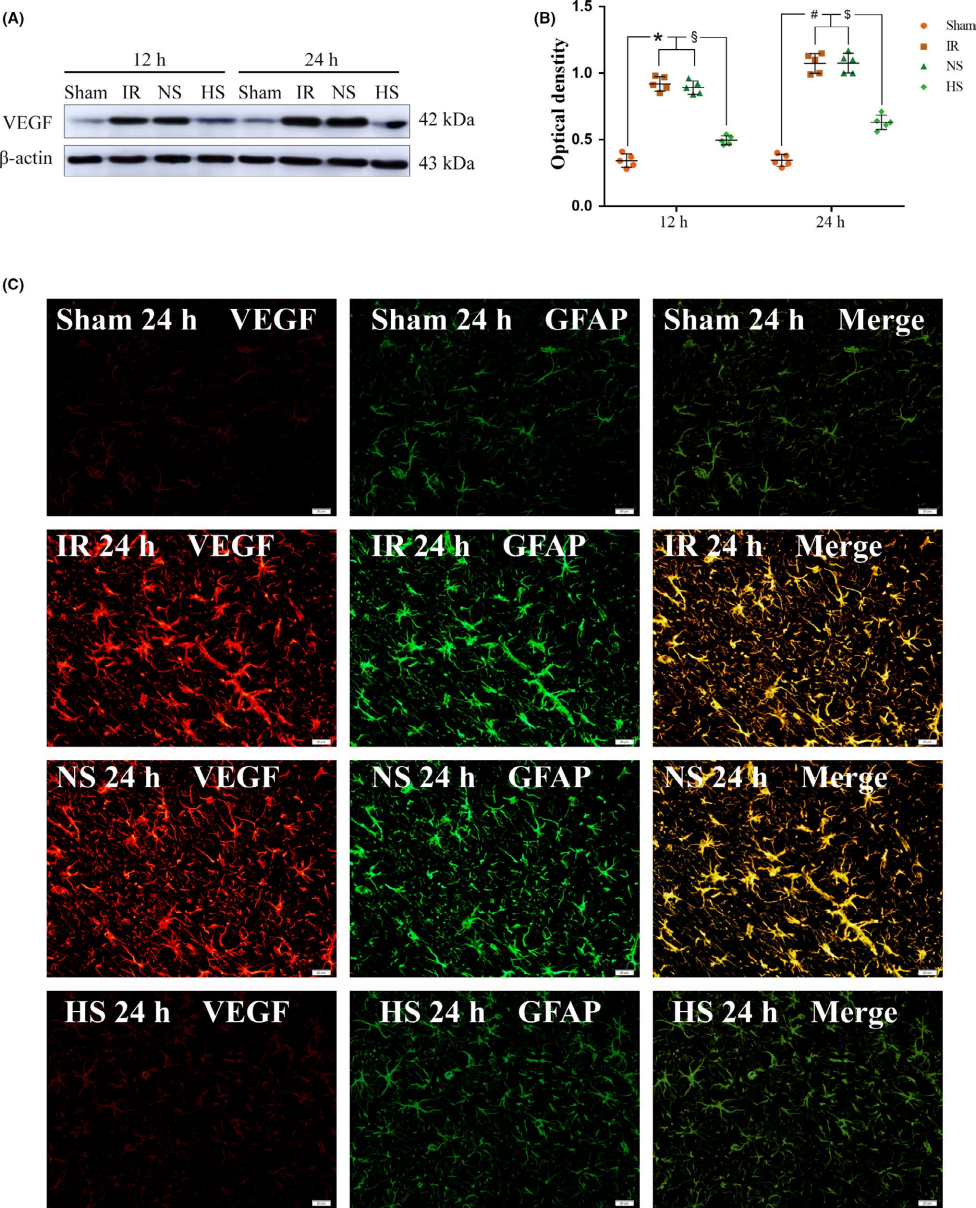
VEGF protein expression in the astrocytes of the peri‐ischemic brain tissue. Panel A shows the VEGF (42 kDa) and β‐actin (43 kDa) immunoreactive bands detected by the primary antibody in the astrocytes of the peri‐ischemic brain tissues at 12 and 24 h in different groups, respectively. B, Compared with the expression levels in the sham group, the expression levels of VEGF protein in peri‐ischemic brain tissues were increased significantly in the IR and NS groups 12 and 24 h after reperfusion, respectively (^*^ *P* < .05 and ^#^ *P* < .05 vs the sham group); Compared with the expression in the IR and NS groups, the expression of the VEGF protein was decreased significantly at 12 and 24 h in the HS group (^§^ *P* < .05 and ^$^ *P* < .05 vs the IR and NS groups). The values represent the means ± SD, n = 5. Panel C shows VEGF immunofluorescence staining (red) in the astrocytes (green) of different groups. Note the colocalized expression of VEGF and GFAP in the astrocytes. Scale bars in panel C: 20 μm. n = 3 for each group

The expression of VEGF protein in peri‐ischemic brain tissue decreased significantly 12 and 24 hours after HS treatment compared to those in the IR and NS groups (^$^ *P* < .05, ^§^ *P* < .05) (Figure 3A,B). The full Western blots of the above each group are shown in Figure S9. Fluorescence imaging showed that compared with those in the sham group, astrocytes in peri‐ischemic brain tissues became proliferative and hypertrophic, and VEGF protein expression in astrocytes increased obviously in the IR and NS groups at 24 hours; however, after HS treatment, the astrocyte proliferative response and VEGF protein expression obviously decreased (Figure 3C).

### 3.4 HS downregulates the expression of IL‐1β protein via inhibiting the activation of the NLRP3 inflammasome in microglia

**FIGURE 4:**
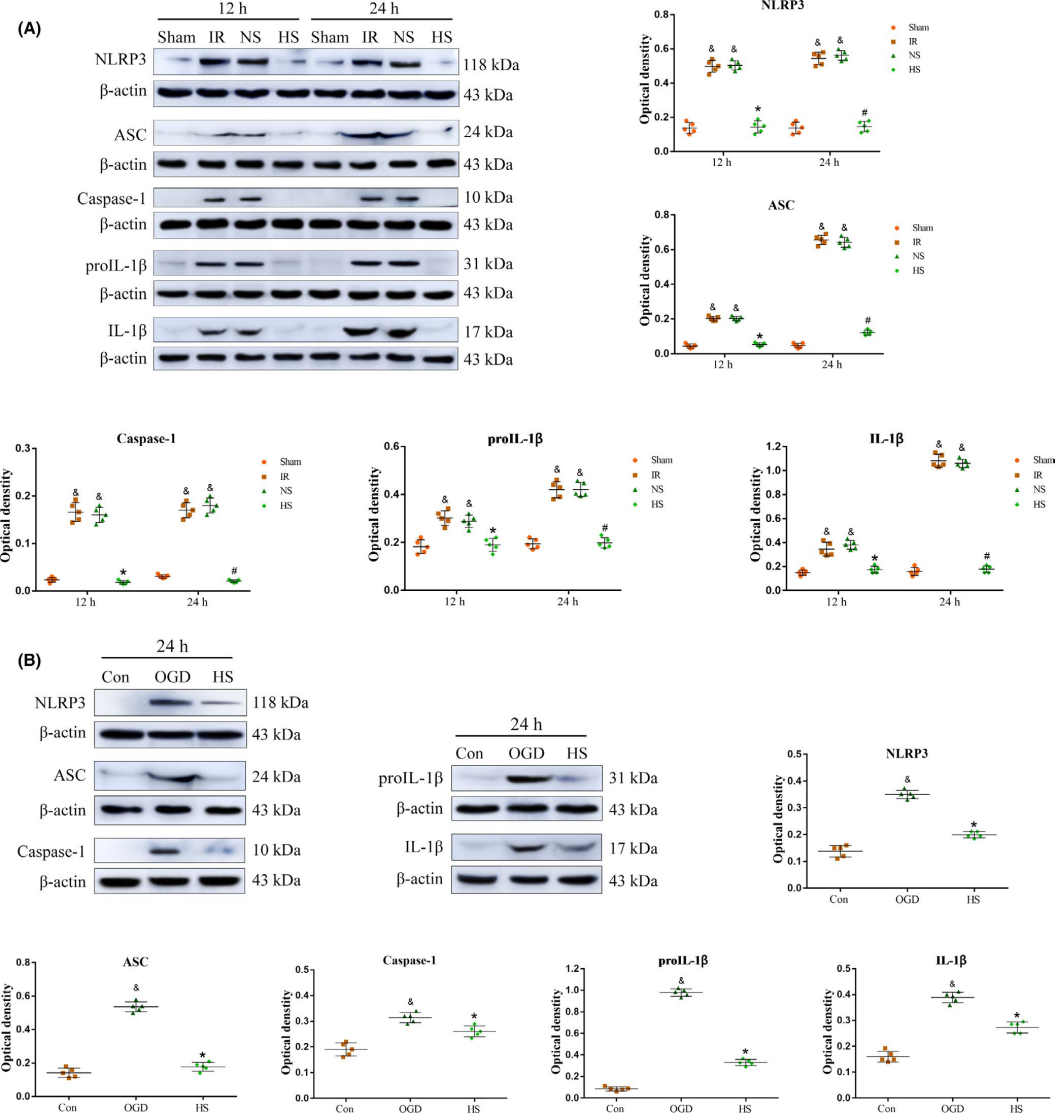
Protein expression of NLRP3, ASC, caspase‐1, pro‐IL‐1β, and IL‐1β in microglia in vivo and in vitro in different groups. A, Compared with the expression levels in the sham group, the expression levels of the NLRP3, ASC, caspase‐1, pro‐IL‐1β, and IL‐1β proteins in the microglia in peri‐ischemic brain tissues were increased significantly in the IR and NS groups 12 and 24 h after reperfusion, respectively (^&^ *P* < .05); however, the expression levels of these proteins were decreased significantly at the corresponding time points after HS treatment (*P < .05, ^#^ *P* < .05). B, The protein expression levels of NLRP3, ASC, caspase‐1, pro‐IL‐1β, and IL‐1β in BV‐2 microglial cells were increased at 24 h in the OGD group compared with the expression levels in the control group (^&^ *P* < .05), but the above protein expression levels decreased 24 h after incubation with 80 mmol/L HS (*P < .05). The dot plots represent the expression changes of the respective markers. Significant differences in the protein levels are expressed as *^#&^ *P* < .05, and the values represent the means ± SD, n = 5. IR 12 h, NS 12 h, IR 24 h, and NS 24 h vs sham 12 h and sham 24 h (^&^ *P* < .05), HS 12 h vs IR 12 h and NS 12 h (*P < .05), HS 24 h vs IR 24 h and NS 24 h (^#^ *P* < .05), OGD 24 h vs Con 24 h (^&^ *P* < .05), HS 24 h vs OGD 24 h (*P < .05)

Upregulated IL‐1β protein expression induced by brain ischemia can induce BBB permeability and aggravate neural injury. Recent studies have shown that the activation of microglia‐derived NLRP3 inflammasomes plays a key role in the over‐expression of IL‐1β. In this study, Western blotting showed that the protein expression of NLRP3, ASC protein with a cysteine protease (caspase)‐activating recruitment domain, caspase‐1, pro‐IL‐1β, and IL‐1β increased significantly in the microglia of peri‐ischemic brain tissue and microglia with OGD intervention compared with that in the corresponding control group (the sham group in vivo or the control group in vitro) (^&^ *P* < .05), but after HS treatment, the expression of the above proteins decreased significantly (*P < .05, ^#^ *P* < .05) (Figure 4A,B). The full Western blots of the above each group are shown in Figure S10. At the same time, immunofluorescence (Figures S1‐S4) showed that branched microglia transformed into amoeboidic phenotype cells under ischemia or hypoxia, and the expression of NLRP3, ASC, caspase‐1, and IL‐1β proteins was upregulated in microglia compared with the expression in the corresponding control group (the sham group in vivo or the control group in vitro), while their expression was downregulated after HS treatment. This result indicates that HS downregulated the expression of IL‐1β protein, which may be associated with inhibiting the activation of the NLRP3 inflammasome in microglia. ELISA showed that the IL‐1β levels in OGD group were much higher than the control group (^&^ *P* < .05), but after HS treatment, the levels decreased significantly (*P < .05) (Figure S5A).

### 3.5 HS downregulates the expression of IL1R1 and pNF‐κBp65 proteins in astrocytes in peri‐ischemic brain tissue

Crosstalk between microglia and astrocytes plays an important role in brain injury and repair. The above results show that HS could inhibit the activation of microglia and downregulate the expression of IL‐1β by inhibiting NLRP3 inflammasome activation in microglia. Thus, HS could affect the expression of IL1R1 and pNF‐Bp65 proteins in astrocytes. Compared with that in the sham group, the expression of IL1R1 and pNF‐Bp65 proteins in peri‐ischemic brain tissue increased significantly in the IR and NS groups 12 and 24 hours after reperfusion (*P < .05, ^#^ *P* < .05); however, the expression of these proteins decreased significantly after HS treatment at the corresponding time (^$^ *P* < .05, ^§^ *P* < .05) (Figure S6A,B and S7). The full Western blots of the above each group are shown in Figure S12 and S13. In addition, double immunofluorescence labeling showed that the expression of the IL1R1 protein in the astrocyte in peri‐ischemic brain tissue was upregulated in the IR and NS groups 24 hours after reperfusion compared with the expression in the sham group, but the expression of IL1R1 was downregulated after HS treatment (Figure S6C). These results indicate that HS can downregulate the expression of IL1R1 and pNF‐κBp65 proteins in astrocytes in peri‐ischemic brain tissue.

### 3.6 HS downregulates the expression of VEGF protein associated with inhibiting the IL‐1β/IL1R1/NF‐κB signaling pathway in astrocytes

**FIGURE 5:**
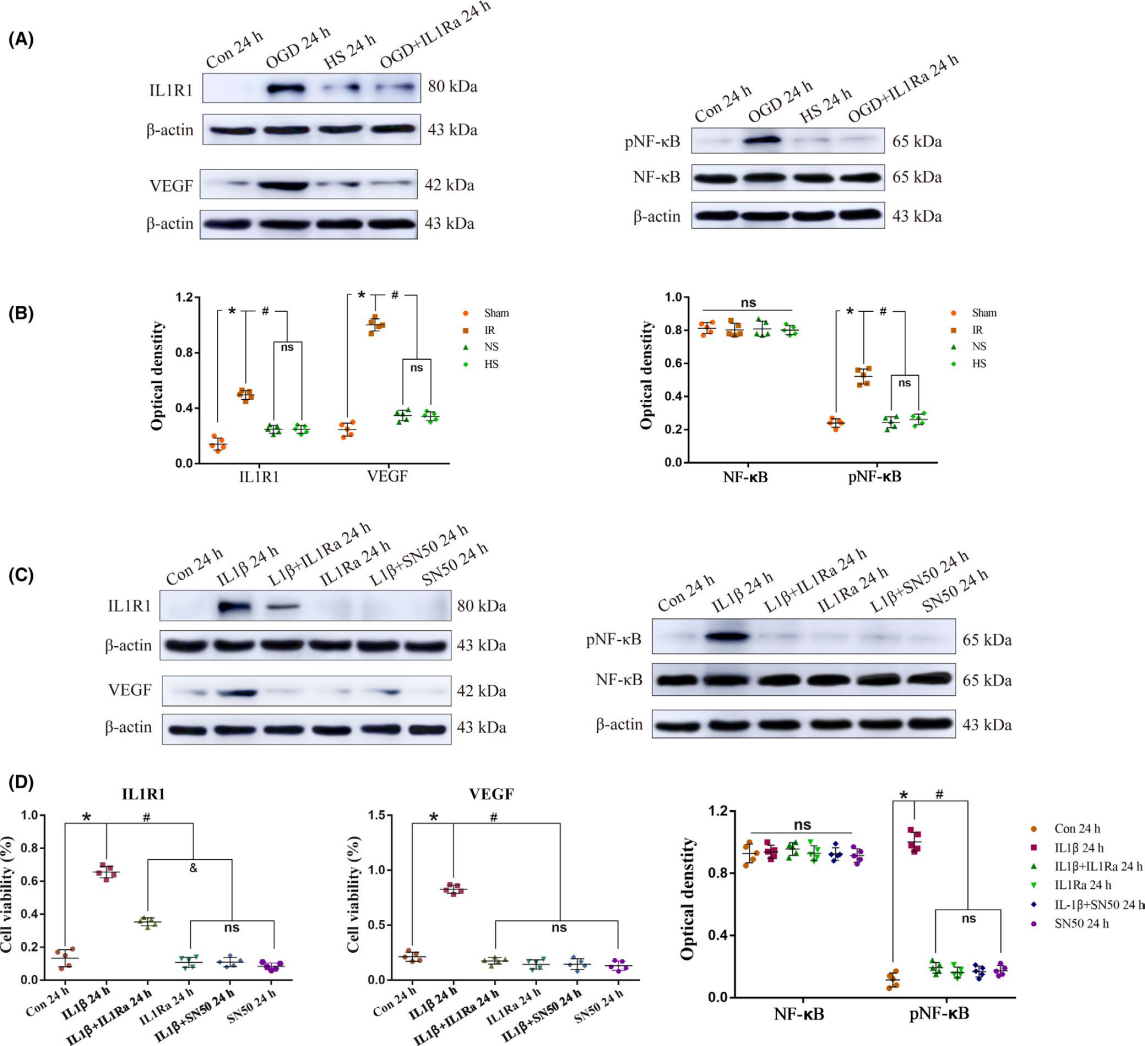
HS downregulates the expression of VEGF protein associated with inhibiting the IL‐1β/IL1R1/NF‐κB signaling pathway in astrocytes. Panels A and C show the IL1R1 (80 kDa), NF‐κBp65 (65 kDa), pNF‐κBp65 (65 kDa), VEGF (42 kDa), and β‐actin (43 kDa) immunoreactive bands detected by the primary antibody in TNC1 astrocytes at 24 h in different groups. B, The protein expression of IL1R1, pNF‐κBp65, and VEGF increased significantly at 24 h in the OGD group compared with that in the control group (*P < .05 vs control group); however, compared with the that in the OGD group, their expression decreased at 24 h in the ILIRa and HS groups (^#^ *P* < .05 vs OGD group). D, The expression of the IL1R1, pNF‐κBp65 and VEGF proteins increased significantly after incubation with recombinant IL‐1β in astrocytes at 24 h compared with that in the control (*P < .05), but their expression decreased at 24 h after incubation with IL1Ra pretreatment and SN50 (^#^ *P* < .05). The values represent the means ± SD, n = 5 for each group; ns: non‐significant, *P* > .05

**FIGURE 6:**
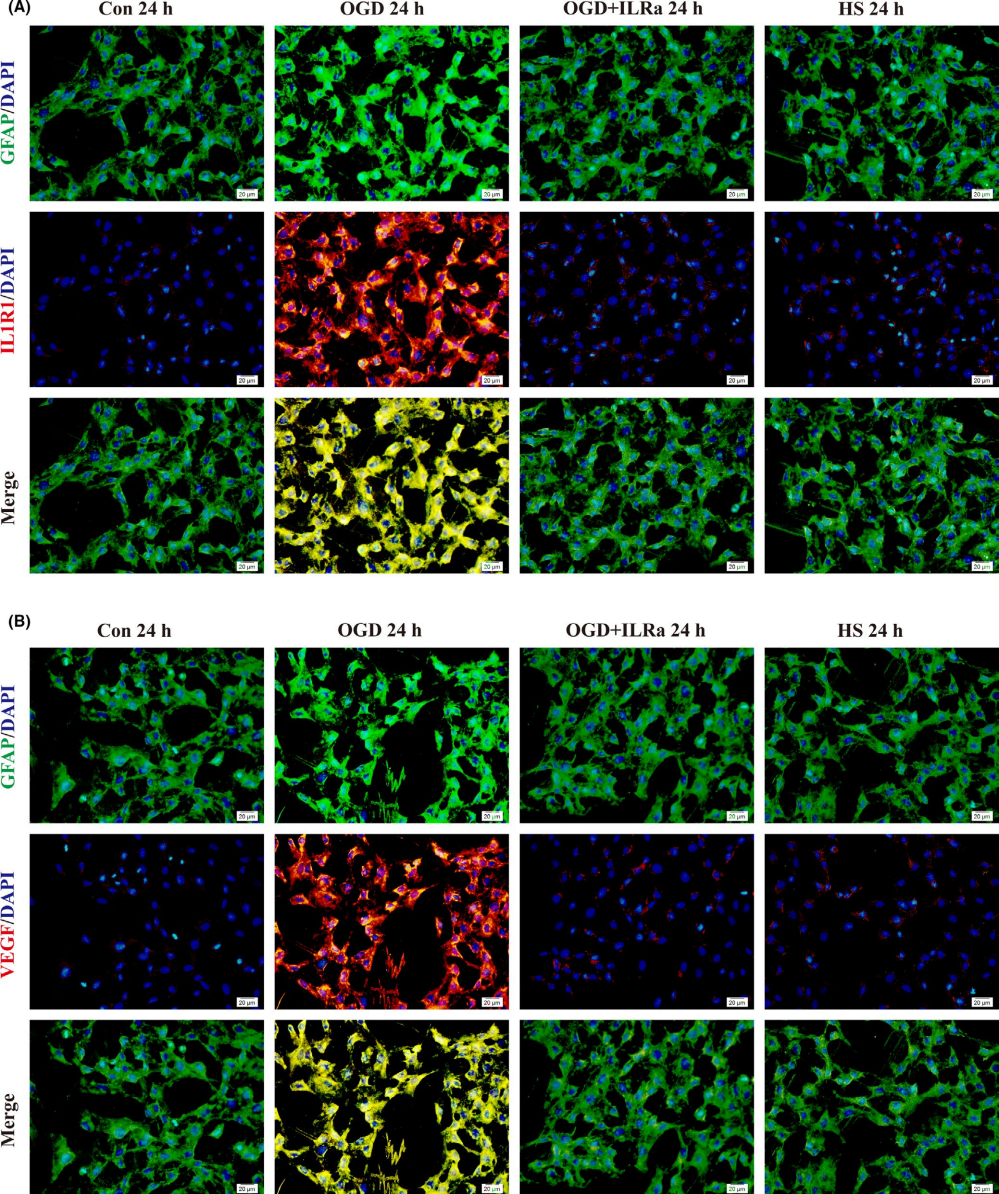
Expression of IL1R1 and VEGF proteins in different groups in TNC1 astrocytes as detected by immunofluorescence. The immunofluorescence images (A) show that compared with that in the control group, the IL1R1 protein expression (red) in GFAP‐positive TNC1 astrocytes (green) was markedly enhanced in the OGD group 24 h after incubation with the culture medium of incubated microglia for hypoxia for 2 h and reoxygenation for 24 h. However, its expression declined at 24 h in the IL1Ra and HS groups. B, Likewise, the VEGF protein expression (red) in the GFAP‐positive TNC1 astrocytes (green) was obviously increased at 24 h in the OGD group compared with that in the control group; however, its expression was decreased in the HS group compared with those in the corresponding groups. Scale bars: 20 µm. DAPI‐blue. n = 3 for each group

To prove that HS downregulated the expression of the VEGF protein, which may be associated with inhibiting the IL‐1β/IL1R1/NF‐κB signaling pathway in astrocytes, astrocytes were incubated with microglia conditioned medium of the control group, the OGD group, and the HS group as mentioned in the above experiment for 24 hours. Based on the above, the IL1Ra + OGD group (IL1Ra group) was added to clarify the above mechanism. The results showed that compared with the expression in the control group, the expression of the ILIR1, pNF‐κBp65, and VEGF proteins in astrocytes increased significantly in the OGD group (*P < .05), but their expression in the IL1Ra and HS groups decreased significantly 24 hours after incubation with the corresponding culture medium (^#^ *P* < .05) (Figure 5A,B). The full Western blots of the above each group are shown in Figure S11A‐C. Moreover, double immunofluorescence showed that HS can downregulate the expression of the ILIR1 and VEGF proteins in astrocytes (Figure 6). HS downregulated the expression of VEGF protein in astrocytes, which may be associated with inhibiting the activation of the IL‐1β/IL1R1/NF‐κB signaling pathway.

### 3.7 Verification that the IL‐1β‐induced upregulated VEGF protein expression in astrocytes is mediated by the IL1R1/NF‐κB signaling pathway in vitro

To verify that IL‐1β upregulates VEGF protein expression in astrocytes and is mediated by the IL1R1/NF‐κB signaling pathway, we used recombinant IL‐1β, IL1Ra, and SN50 (an NF‐κBp65 inhibitor) to incubate the astrocytes for 24 hours in vitro. Western blotting showed that compared with the expression in the control group, the expression of the IL1R1, pNF‐κBp65, and VEGF proteins increased significantly in astrocytes 24 hours after incubation with recombinant IL‐1β, but their expression decreased significantly after incubation with IL1Ra pretreatment and SN50 (Figure 5C,D). The full Western blots of the above each group are shown in Figure S11D‐F.

## 4 DISCUSSION

In this study, we found that 10% HS can reduce the release of IL‐1β by inhibiting the activation of the NLRP3 inflammasome in microglia and then downregulates the astrocyte‐derived VEGF expression by inhibiting the activity of the IL‐1β/IL1R1/NF‐кB signaling pathway in astrocytes in focal ischemic stroke in rats. As a result, HS alleviates BBB permeability induced by ischemic stroke through the above mechanism, which may be an anti‐edema mechanism for HS to treat ischemic brain edema and ICP.

It is well documented that the neuroinflammatory response plays an important role in ischemic brain injury. Recent studies have shown that DAMPs activate the neuroimmune inflammatory response through Toll‐like receptor and/or NOD‐like receptor signaling pathways in ischemic stroke.^30^ Thus, a large number of inflammatory mediators are released from microglia, astrocytes, and neurons, which aggravate brain injury. In recent years, many studies have found that microglia‐derived NLRP3 inflammasomes play a key role in initiating the neuroinflammatory response by releasing IL‐1β, TNF‐α, MCP‐1, etc The activation of microglia‐derived NLRP3 inflammasomes can upregulate the expression of IL‐1β and IL‐18 and can lead to inflammatory cascades mediated by the paracrine secretion or autocrine secretion of IL‐1β and exacerbate cerebral injury.

Hypertonic saline alleviates brain edema and reduces ICP caused by multiple etiologies, except for the osmotic dehydration mechanism; recent studies have found that HS has non‐osmotic dehydration mechanisms, including exerting antiinflammatory effects,^25^ downregulating the AQP4 expression in astrocytes,^31^ and reducing the permeability of BBB by downregulating astrocyte‐derived VEGF.^8^ It is well documented that HS has an antiinflammatory effect and exerts the function of organ protection induced by trauma‐hemorrhagic shock,^32^, ^33^, ^34^, ^35^ acute pancreatitis,^36^ traumatic brain injury,^31^ and ischemic stroke.^25^ Studies have shown that HS can alleviate acute lung injury and the intestinal mucosa barrier function induced by hemorrhagic shock by inhibiting neutrophil activation and by reducing neutrophil (PMN)‐endothelial (EC) adhesion and the expression of pro‐inflammatory cytokines, including TNF‐ɑ, IL‐1β, and IL‐6.^33^, ^34^, ^37^, ^38^, ^39^, ^40^ An animal experiment found that HS may ameliorate the brain edema and brain injury induced by traumatic brain injury by reducing TNF‐ɑ‐ and IL‐1β‐mediated pro‐inflammatory activation.^25^, ^31^ In this study, the in vitro and in vivo results show that HS can reduce the activation of the NLRP3 inflammasome and its adaptor protein apoptosis‐associated speck‐like protein (ASC) in microglia. As a result, the expression levels of the caspase‐1 and IL‐1β proteins were downregulated. Thus, the mechanism by which HS reduces IL‐1β secretion may be related to the inhibition of the NLRP3/IL‐1β signaling pathway in microglia, which may be associated with playing a role in brain protection in transient focal cerebral ischemia. It has been reported that high salt induces severe inflammatory reactions,^41^, ^42^, ^43^ and NLRP3 inflammasome is activated under hypertonic conditions.^44^, ^45^, ^46^ However, there was no inflammation in all the study subjects before being treated with high salt or hypertonic conditions. In the present study, NLRP3 inflammasome was activated by ischemia before being treated with HS. Indeed, we have reported that HS noticeably decreased TNF‐α and IL‐1β expression in ischemic microglia.^25^

It has been shown that crosstalk between microglia and astrocytes can regulate the function of astrocytes to aggravate nerve injury and repair.^22^, ^23^, ^24^ A study found that astrocyte activation was mediated by activated microglia in vitro and in vivo, which induced astrocytic hyperplasia and hypertrophy and enhanced the protein expression of TNF‐α, IL‐1β, and iNOS.^24^ It has been documented that the capacity to form a functional NLRP3 inflammasome and to secrete IL‐1β is limited to the microglial compartment in the mouse brain but is not limited in astrocytes, thus indicating that microglia‐dependent inflammasome activation can play an important role in the brain, especially in neuroinflammatory conditions.^13^ It has been shown that IL‐1β is one of the major inflammatory factors that induces ischemic brain injury, which increases BBB permeability, neuron death, and even the transformation of cerebral hemorrhage in ischemic stroke.^47^, ^48^ In this study, our results show that the expression of IL‐1R1 and VEGF in astrocytes and pNF‐кBp65 in peri‐ischemic brain tissue was upregulated in the IR group compared with the expression in the sham group, while their expression was downregulated after HS treatment. It was further confirmed that the expression of IL‐1R1, pNF‐кBp65 and VEGF in astrocytes was upregulated after incubation with microglial culture medium after OGD intervention, while their expression was downregulated after incubation with IL1Ra or 80 mmol/L HS in vitro. At the same time, it was verified in vitro that the expression of astrocyte‐derived VEGF induced by IL‐1β was associated with the activation of the IL‐1R1/NF‐кB pathway. These results indicate that HS can downregulate astrocyte‐derived VEGF protein via inhibiting the activation of the IL‐1R1/NF‐кB signal transduction axis. It was shown that IL‐1β can induce VEGF expression in a time‐ and concentration‐dependent manner and can increase both VEGF promoter activity and mRNA half‐life in vascular smooth muscle cells.^49^ The induction of VEGF gene transcription by IL‐1β is mediated through stress‐activated MAP kinases and Sp1 sites in cardiac myocytes.^50^ Another study showed that upregulating VEGF protein by IL‐1β may be related to activating the hypoxia‐inducible factor‐1 (HIF‐1)–responsive gene VEGF via a pathway that is dependent on NF‐кB.^51^

It is well documented that activated microglia secrete an array of pro‐inflammatory cytokines including TNF‐α and IL‐1β, and both are involved in disruption of BBB.^52^, ^53^, ^54^, ^55^ However, the lack of other cytokines (ie, TNF‐a) being investigated in the present study is a limitation and an area for future research. Of course, our study did not prove the upstream mechanism by which HS inhibits NLRP3 inflammasome activation in ischemic stroke. It is well documented that the disruption of K^+^ is mediated by P2X purinoceptor 7 and Ca^2+^ homeostasis, and mtDNA and mtROS release from mitochondrial dysfunction and lysosomal rupture have been demonstrated to have important roles in the activation of the NLRP3 inflammasome.^56^ It will be further confirmed whether HS exerts anti‐edemic effects and alleviates BBB permeability in ischemic brain injury. In addition, the expression of IL‐1R1, pNF‐кB, and VEGF in astrocytes was upregulated after incubation with microglial culture medium after OGD intervention, while their expression was downregulated after incubation with IL1Ra or 80 mmol/L HS in vitro. It seems HS is able to reduce VEGF production in astrocyte via decreasing IL‐1β expression in ischemic microglia, and via blocking IL‐1β/IL‐1R1/pNF‐кB signaling pathway in astrocyte. However, the mechanism of upregulating VEGF mediated by NF‐кB is unclear in astrocytes in ischemic injury, and it is unclear if this association is mediated by the hypoxia‐inducible factors reported in a previous study.^51^

## 5 CONCLUSIONS

10% HS can alleviate BBB permeability induced by cerebral ischemia via downregulating the astrocyte‐derived VEGF protein in focal ischemic stroke in rats; the mechanism by which this occurs may involve HS reducing the release of IL‐1β by inhibiting the activation of the NLRP3 inflammasome in microglia and then downregulating the astrocyte‐derived VEGF expression through the IL‐1β/IL1R1/NF‐кB signaling pathway in astrocytes.

## CONFLICT OF INTEREST

The authors declare no conflict of interest.

## Supporting information

Fig S1‐S13
