# Supplementary material for: Hypertonic saline mediates the NLRP3/IL‐1β signaling axis in microglia to alleviate ischemic blood‐brain barrier permeability by downregulating astrocyte‐derived VEGF in rats
Source: CNS Neurosci Ther. 2020 Jun 12;26(10):1045–57. doi: 10.1111/cns.13427 (PMC7539845; doi:10.1111/cns.13427)

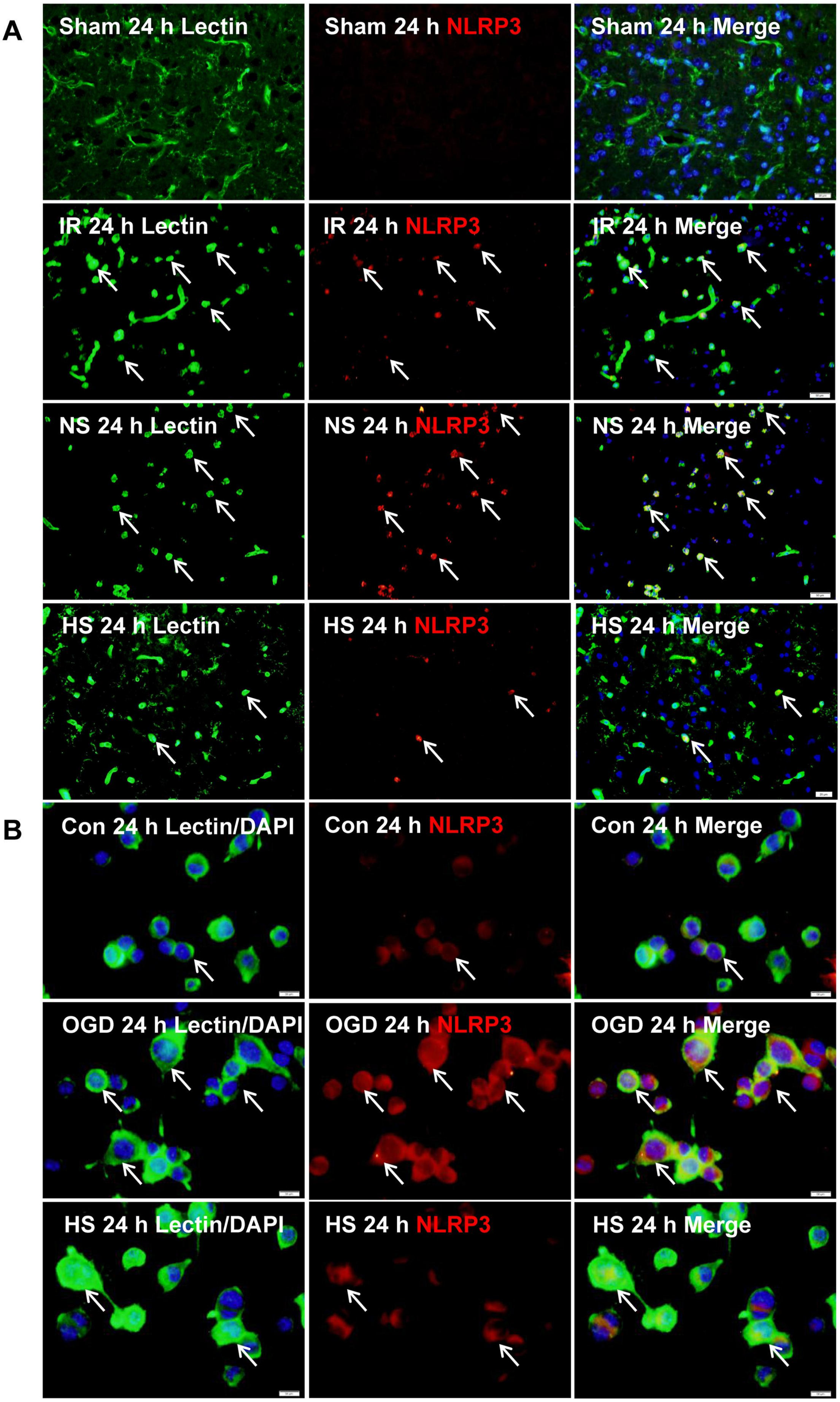
A
Sham 24 h Lectin
Sham 24 h NLRP3
Sham 24 h Merge
IR 24 h Lectin
IR 24 h NLRP3
IR 24 h Merge
NS 24 h Lectin
NS 24 h NLRP3
NS 24 h Merge
HS 24 h Lectin
HS 24 h NLRP3
HS 24 h Merge
B
Con 24 h Lectin/DAPI
Con 24 h NLRP3
Con 24 h Merge
OGD 24 h Lectin/DAPI
OGD 24 h NLRP3
OGD 24 h Merge
HS 24 h Lectin/DAPI
HS 24 h NLRP3
HS 24 h Merge

**Supplementary Figure 1** HS downregulates NLRP3 protein expression in microglia in ischaemia-reperfusion or hypoxia-reoxygenation as detected by immunofluorescence. The immunofluorescence images (A) show that compared with that in the sham group, NLRP3 protein expression (red) in lectin-positive microglia (green) in the peri-ischaemic brain tissue was markedly enhanced in the IR and NS groups 24 h after reperfusion, but its expression declined 24 h after HS treatment. B. Likewise, the *in vitro* NLRP3 protein expression (red) in lectin-positive BV-2 microglial cells (green) was obviously increased at 24 h in the OGD group compared with that in the control group; however, its expression decreased after incubation with 80 mM HS for 24 h. Scale bars: 20 μm. DAPI-blue. n=3 for each group.

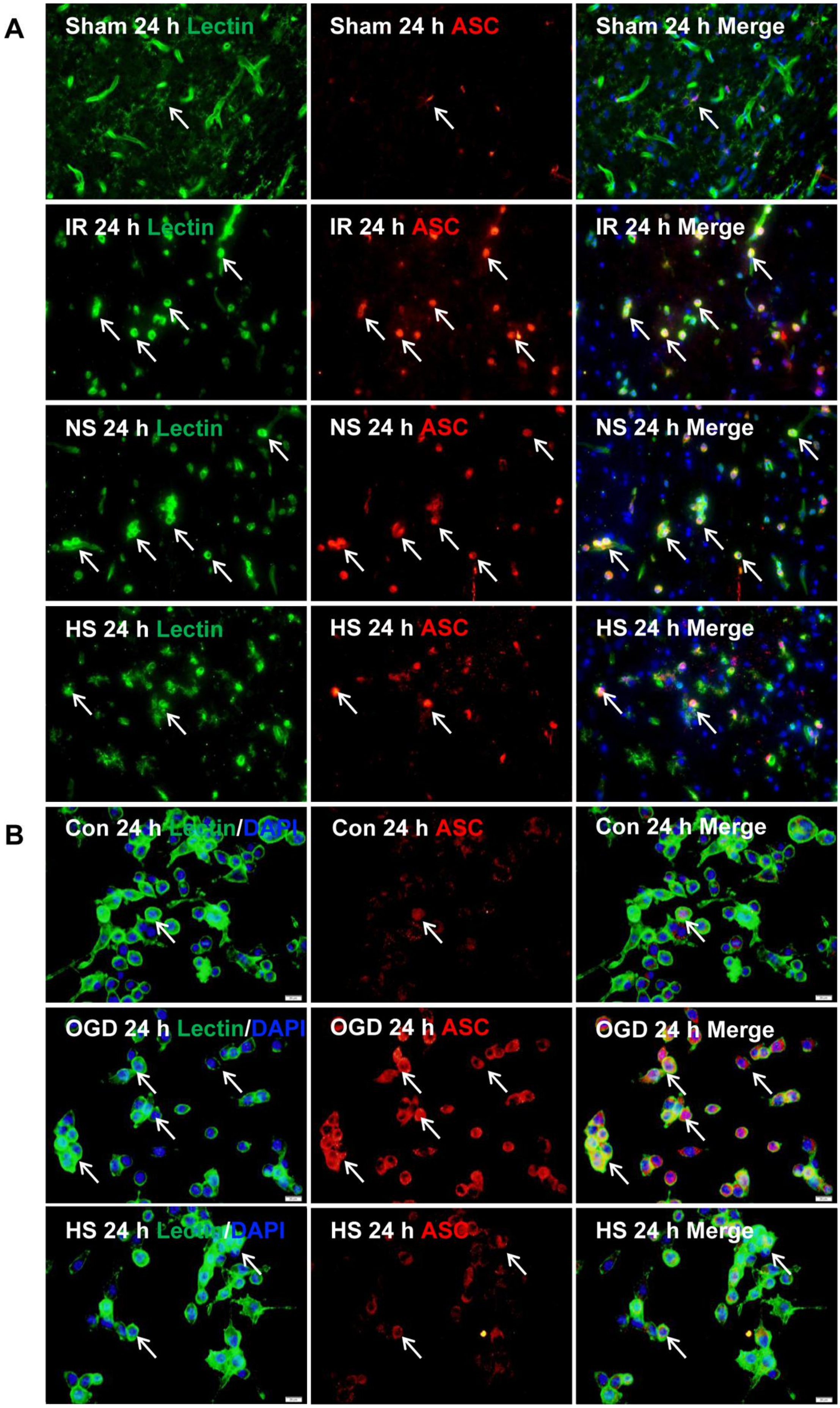
A
Sham 24 h Lectin
Sham 24 h ASC
Sham 24 h Merge
IR 24 h Lectin
IR 24 h ASC
IR 24 h Merge
NS 24 h Lectin
NS 24 h ASC
NS 24 h Merge
HS 24 h Lectin
HS 24 h ASC
HS 24 h Merge
B
Con 24 h Lectin/DAPI
Con 24 h ASC
Con 24 h Merge
OGD 24 h Lectin/DAPI
OGD 24 h ASC
OGD 24 h Merge
HS 24 h Lectin/DAPI
HS 24 h ASC
HS 24 h Merge

**Supplementary Figure 2** HS downregulates ASC protein expression in microglia in ischaemia-reperfusion or hypoxia-reoxygenation as detected by immunofluorescence. The immunofluorescence images (A) show that compared with that in the sham group, ASC protein expression (red) in lectin-positive microglia (green) in the peri-ischaemic brain tissue was markedly enhanced in the IR and NS groups 24 h after reperfusion, but its expression declined at 24 h after HS treatment. B. Likewise, the *in vitro* ASC protein expression (red) in lectin-positive BV-2 microglial cells (green) was obviously increased at 24 h in the OGD group compared with that in the control group; however, its expression was decreased after incubation with 80 mM HS for 24 h. Scale bars: 20 μm. DAPI-blue. n=3 for each group.

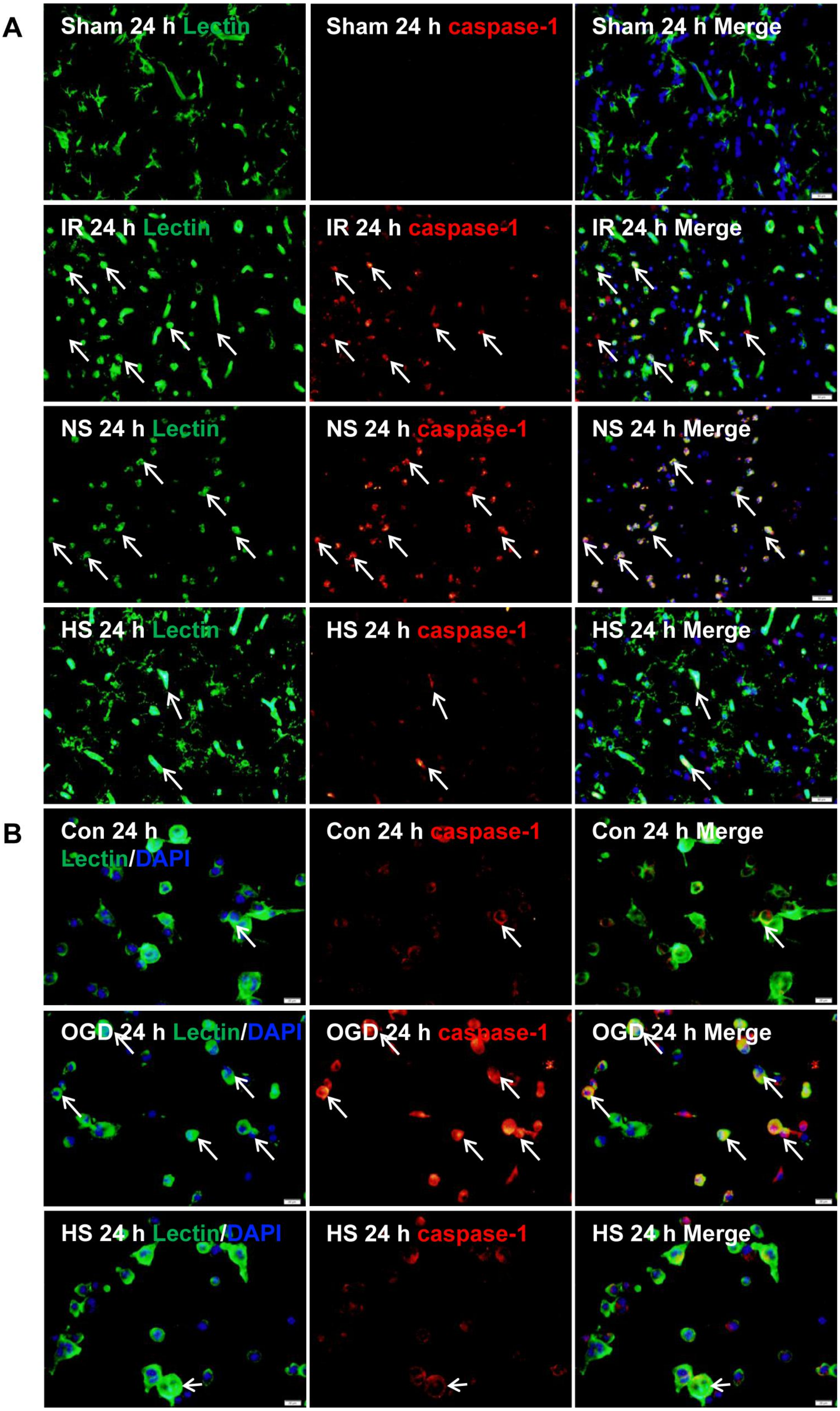

A
Sham 24 h Lectin
Sham 24 h caspase-1
Sham 24 h Merge
IR 24 h Lectin
IR 24 h caspase-1
IR 24 h Merge
NS 24 h Lectin
NS 24 h caspase-1
NS 24 h Merge
HS 24 h Lectin
HS 24 h caspase-1
HS 24 h Merge
B
Con 24 h Lectin/DAPI
Con 24 h caspase-1
Con 24 h Merge
OGD 24 h Lectin/DAPI
OGD 24 h caspase-1
OGD 24 h Merge
HS 24 h Lectin/DAPI
HS 24 h caspase-1
HS 24 h Merge

**Supplementary Figure 3** HS downregulates caspase-1 protein expression in microglia in ischaemia-reperfusion or hypoxia-reoxygenation as detected by immunofluorescence. The immunofluorescence images (A) show that compared with that in the sham group, caspase-1 protein expression (red) in lectin-positive microglia (green) in the peri-ischaemic brain tissue was markedly enhanced in the IR and NS groups 24 h after reperfusion, but its expression declined 24 h after HS treatment. B. Likewise, the *in vitro* caspase-1 protein expression (red) in lectin-positive BV-2 microglial cells (green) was obviously increased at 24 h in the OGD group compared with that in the control group; however, its expression was decreased after incubation with 80 mM HS for 24 h. Scale bars: 20 μm. DAPI-blue. n=3 for each group.

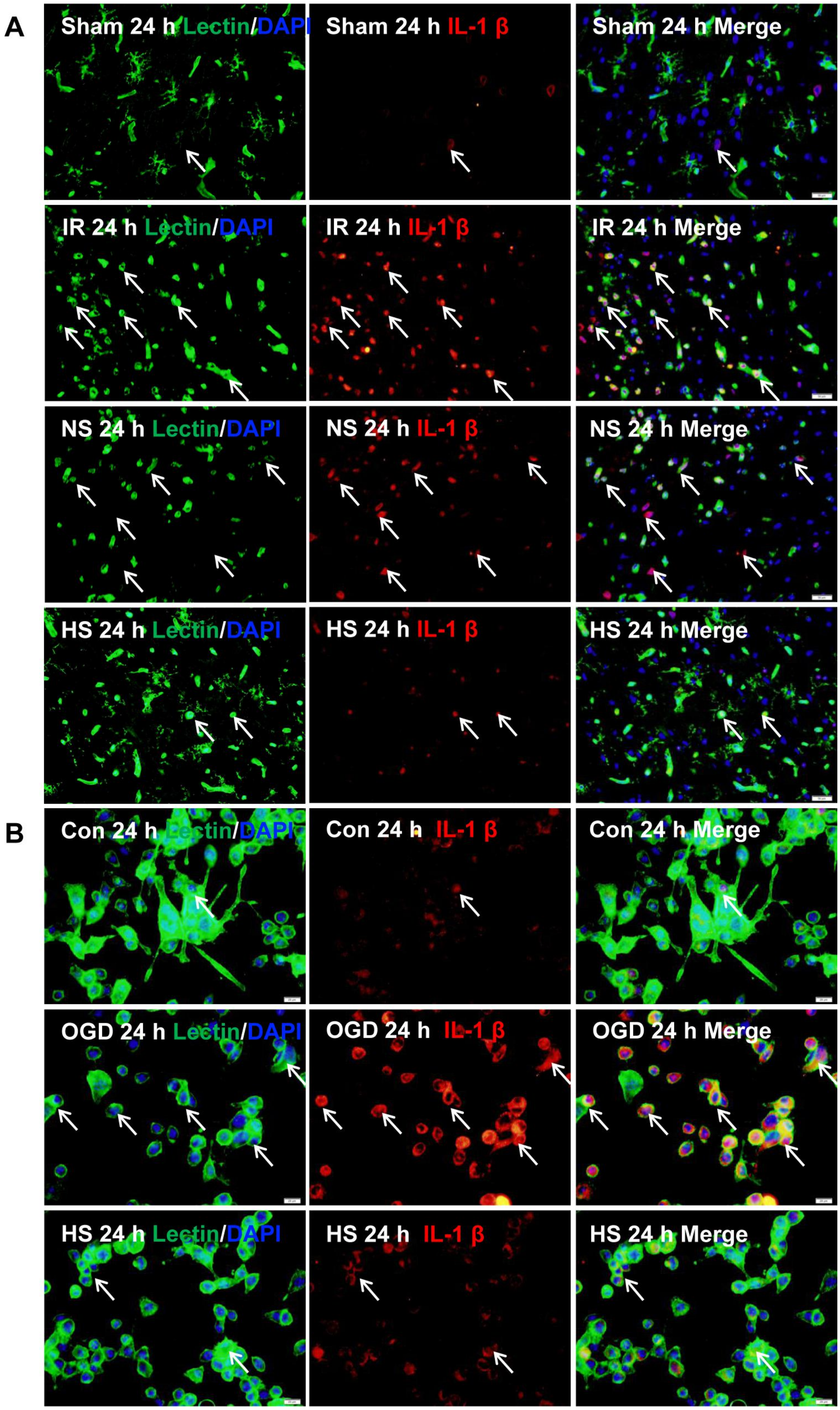
A
Sham 24 h Lectin/DAPI
Sham 24 h IL-1 β
Sham 24 h Merge
IR 24 h Lectin/DAPI
IR 24 h IL-1 β
IR 24 h Merge
NS 24 h Lectin/DAPI
NS 24 h IL-1 β
NS 24 h Merge
HS 24 h Lectin/DAPI
HS 24 h IL-1 β
HS 24 h Merge
B
Con 24 h Lectin/DAPI
Con 24 h IL-1 β
Con 24 h Merge
OGD 24 h Lectin/DAPI
OGD 24 h IL-1 β
OGD 24 h Merge
HS 24 h Lectin/DAPI
HS 24 h IL-1 β
HS 24 h Merge

**Supplementary Figure 4** HS downregulates IL-1β protein expression in microglia in ischaemia-reperfusion or hypoxia-reoxygenation as detected by immunofluorescence. Immunofluorescence images (A) show that compared with that in the sham group, IL-1β protein expression (red) in lectin-positive microglia (green) in the peri-ischaemic brain tissue was markedly enhanced in the IR and NS groups 24 h after reperfusion, but its expression declined 24 h after HS treatment. B. Likewise, the *in vitro* IL-1β protein expression (red) in lectin-positive BV-2 microglial cells (green) was obviously increased at 24 h in the OGD group compared with that in the control group; however, its expression was decreased after incubation with 80 mM HS for 24 h. Scale bars: 20 μm. DAPI-blue. n=3 for each group.

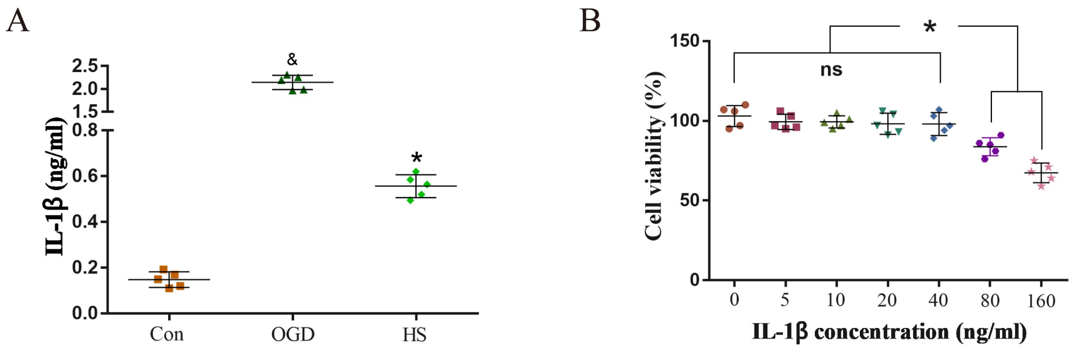


**Supplementary Figure 5** HS downregulates IL-1β protein expression in microglia by ELISA, and the effect of IL-1β on activity of astrocytes. A. The IL-1β levels in OGD group was much higher than the control group ($^{\&}P < 0.05$), but after HS treatment, the levels decreased significantly ($^{*}P<0.05$). The values represent the means ± SD, n=5. B. Appropriate concentrations of IL-1β were detected by the CCK-8 (cell counting kit-8) assay. The viability of TNC1 astrocytes decreased significantly when incubated with IL-1β at a dose exceeding 40 ng/mL. ELISA, enzyme-linked immunosorbent assay.

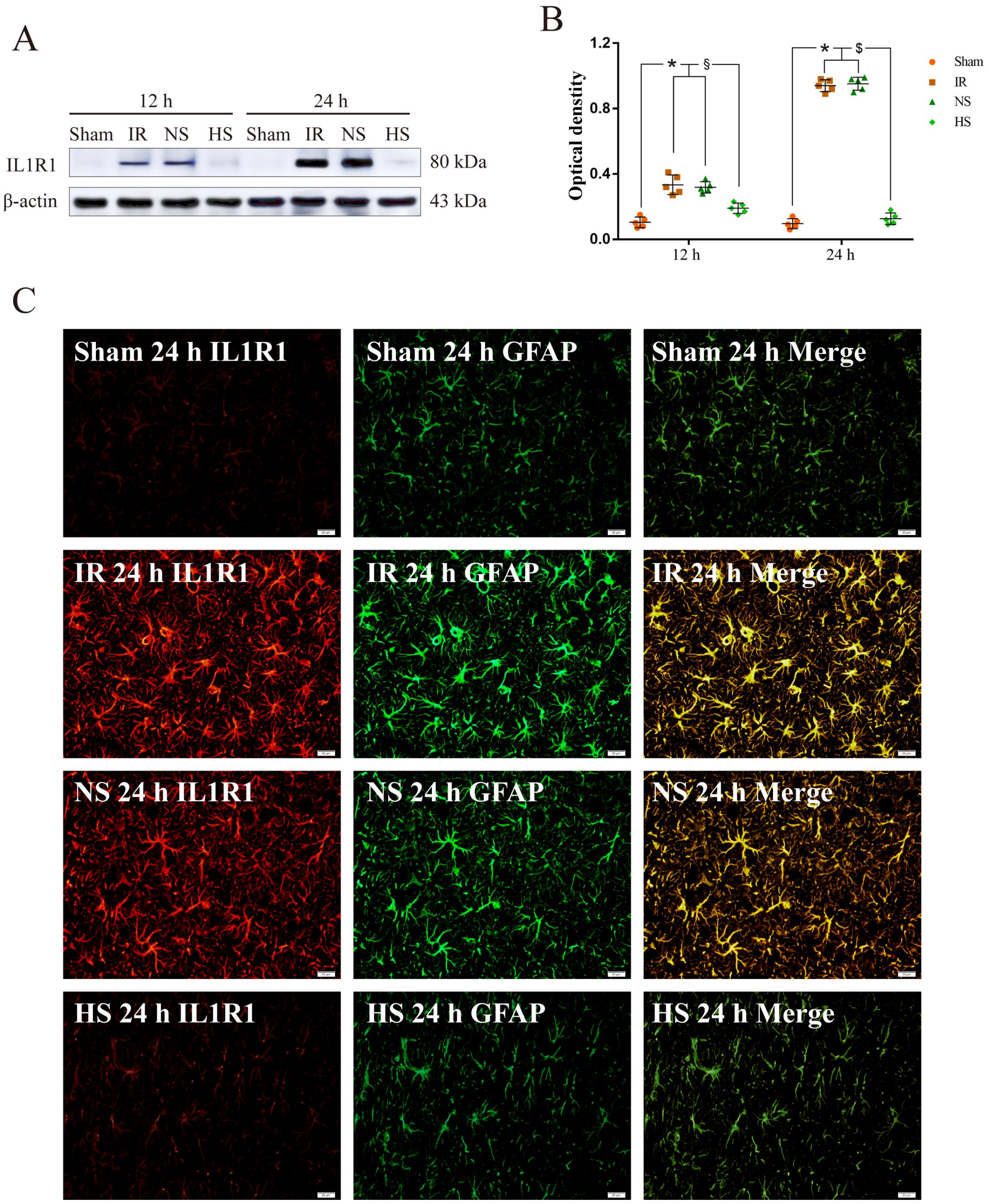
A
12 h
24 h
Sham
IR
NS
HS
Sham
IR
NS
HS
IL1R1
β-actin
80 kDa
43 kDa
B
Optical density
1.2
0.8
0.4
0.0
12 h
24 h
*
§
*
$
Sham
IR
NS
HS
C
Sham 24 h IL1R1
Sham 24 h GFAP
Sham 24 h Merge
IR 24 h IL1R1
IR 24 h GFAP
IR 24 h Merge
NS 24 h IL1R1
NS 24 h GFAP
NS 24 h Merge
HS 24 h IL1R1
HS 24 h GFAP
HS 24 h Merge

**Supplementary Figure 6** IL1R1 protein expression in the astrocytes of the peri-ischaemic brain tissue. Panel A shows the IL1R1 (80 kDa) and β-actin (43 kDa) immunoreactive bands detected by the appropriate primary antibodies in the astrocytes of the peri-ischaemic brain tissue at 12 h and 24 h in different groups, respectively. The bar graph in B shows the downregulation of IL1R1 protein expression at 12 h and 24 h in the HS group compared with the protein expression in the IR and NS groups, respectively ($^{\S}P<0.05$ and $^{\&}P<0.05$ versus the IR and NS groups). The values represent the means ± SD, n=5. Panel C shows IL1R1 immunofluorescence staining (red) in GFAP-positive astrocytes (green) in different groups. Note the colocalized expression of IL1R1 and GFAP in the astrocytes. Scale bars in panel A: 20 μm. n=3 for each group.

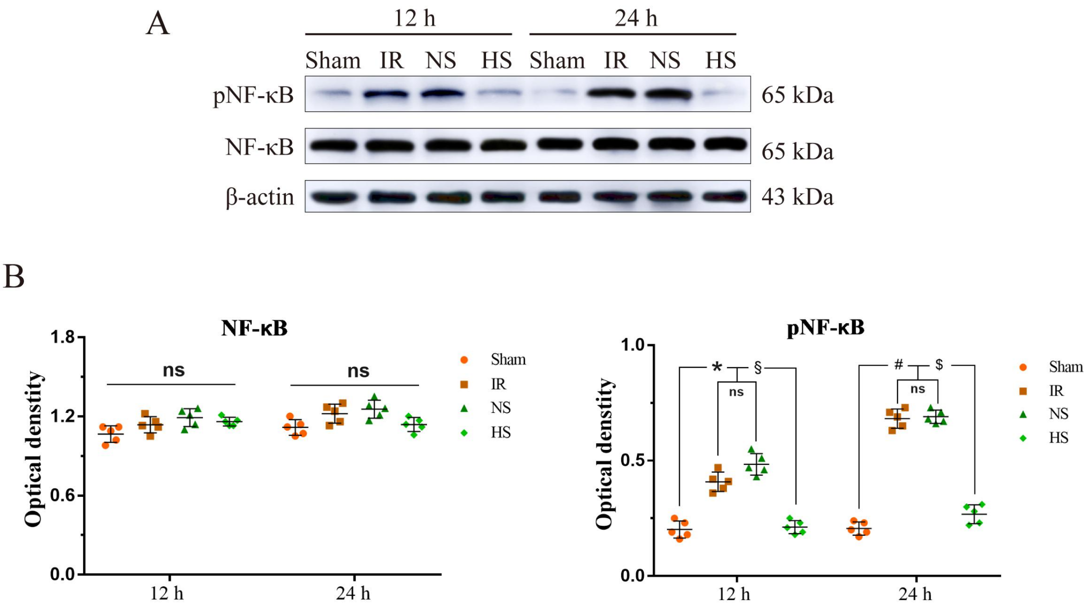


**Supplementary Figure 7** pNF-κBp65 protein expression in the astrocytes of the peri-ischaemic brain tissue. Panel A shows the NF-κBp65 and pNF-κBp65 (65 kDa) and β-actin (43 kDa) immunoreactive bands detected by the primary antibody in the astrocytes of the peri-ischaemic brain tissue at 12 h and 24 h in different groups, respectively. The bar graph in B shows that NF-κBp65 protein expression was not different in each group; however, pNF-κBp65 protein expression at 12 h and 24 h in the HS group was upregulated compared with that in the IR and NS groups ($^{\S}P$<0.05 and $^{\&}P$<0.05 versus IR group and NS group). The values represent the means ± SD, n=5 for each group; ns: non-significant, $P$ > 0.05.

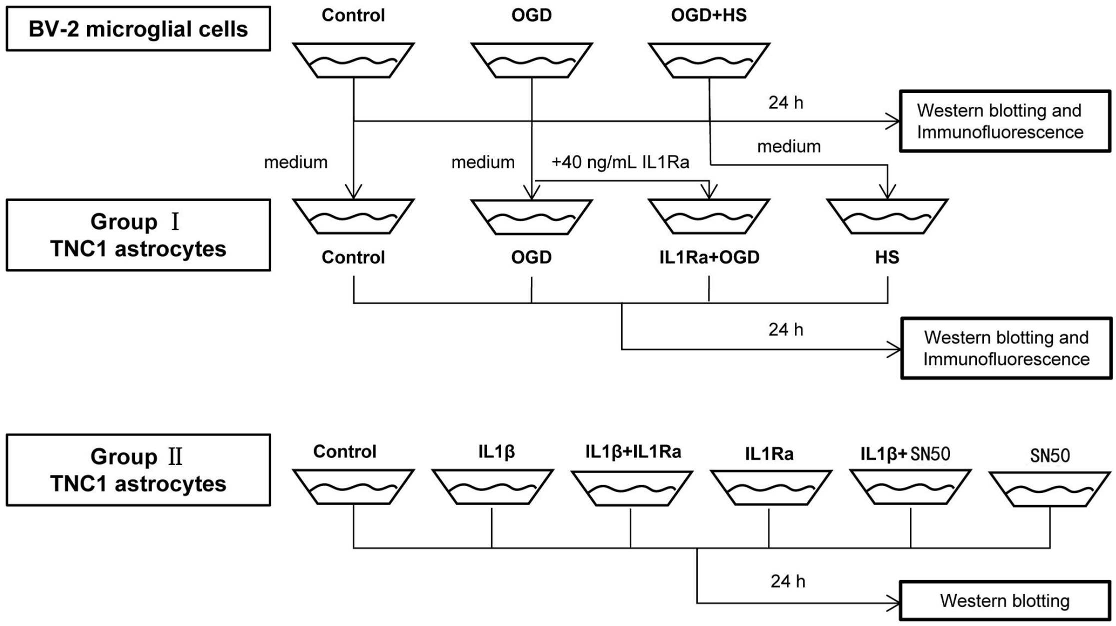


**Supplementary Figure 8** The schematic diagram depicts the design and flow chart of the cell experiment. The schematic diagram depicts the design and flow chart of the cell experiment *in vitro*.

**Supplementary Figure 9** Full unedited blots for Figure 3.

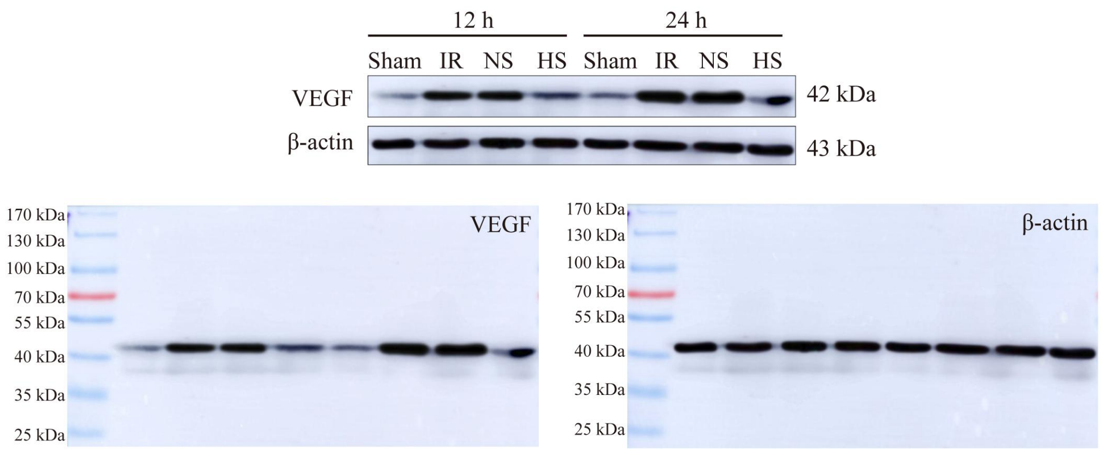


**Supplementary Figure 10** Full unedited blots for Figure 4.

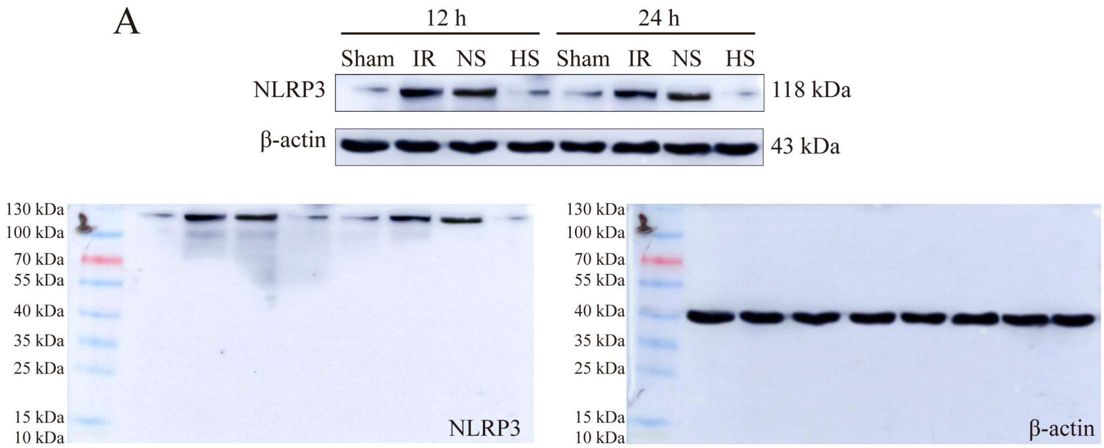

B

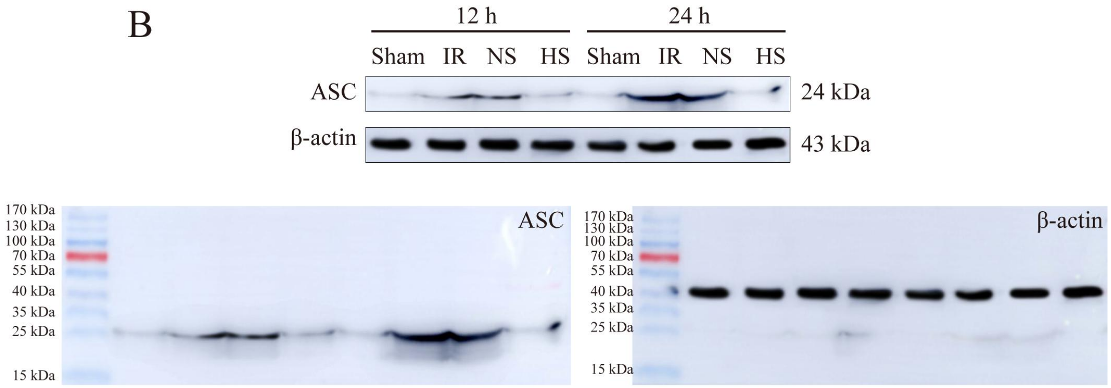
12 h
24 h
Sham IR NS HS Sham IR NS HS
ASC
24 kDa
β-actin
43 kDa
170 kDa
130 kDa
100 kDa
70 kDa
55 kDa
40 kDa
35 kDa
25 kDa
15 kDa
ASC
β-actin

C

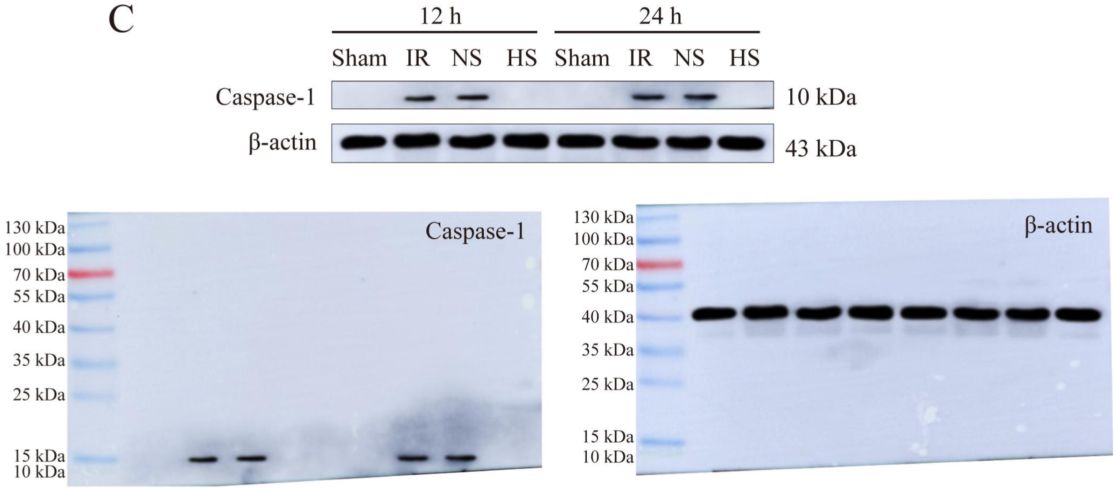
12 h
24 h
Sham IR NS HS Sham IR NS HS
Caspase-1
10 kDa
β-actin
43 kDa
130 kDa
100 kDa
70 kDa
55 kDa
40 kDa
35 kDa
25 kDa
15 kDa
10 kDa
Caspase-1
β-actin

D

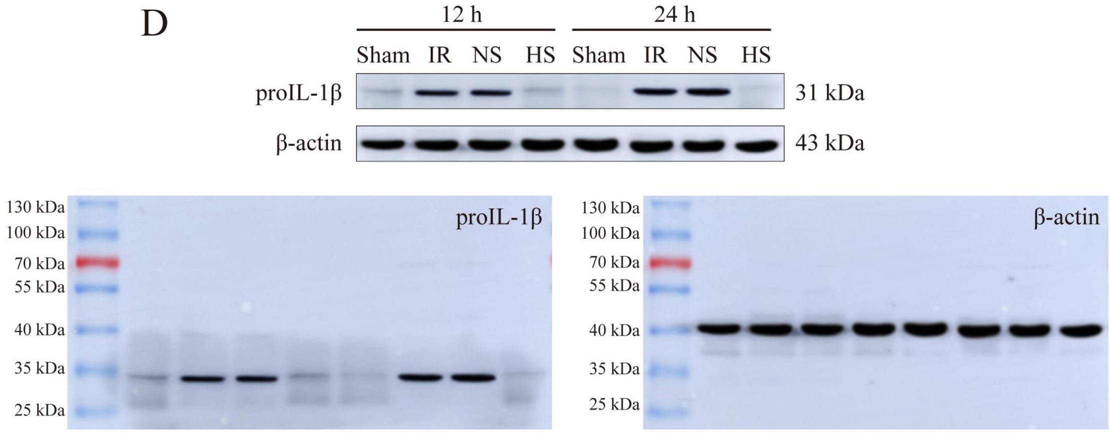
12 h
24 h
Sham IR NS HS Sham IR NS HS
proIL-1β
31 kDa
β-actin
43 kDa
130 kDa
100 kDa
70 kDa
55 kDa
40 kDa
35 kDa
25 kDa
proIL-1β
β-actin

E

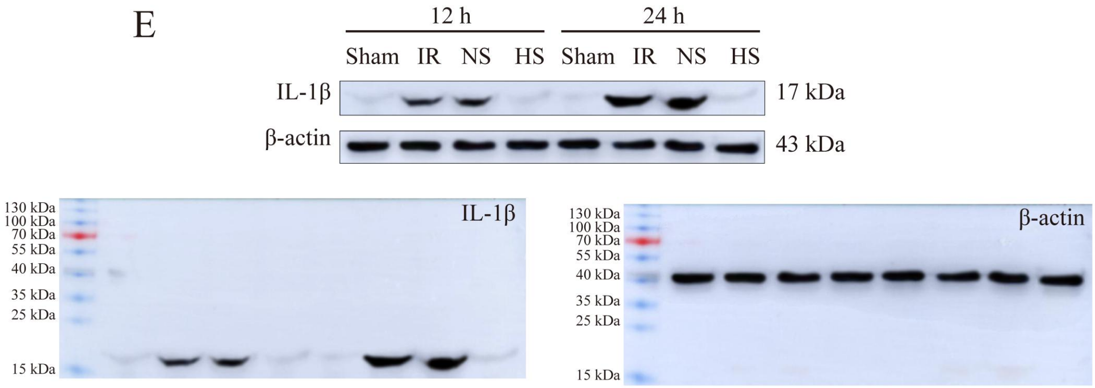
12 h
24 h
Sham IR NS HS Sham IR NS HS
IL-1β
17 kDa
β-actin
43 kDa
130 kDa
100 kDa
70 kDa
55 kDa
40 kDa
35 kDa
25 kDa
15 kDa
IL-1β
β-actin

F

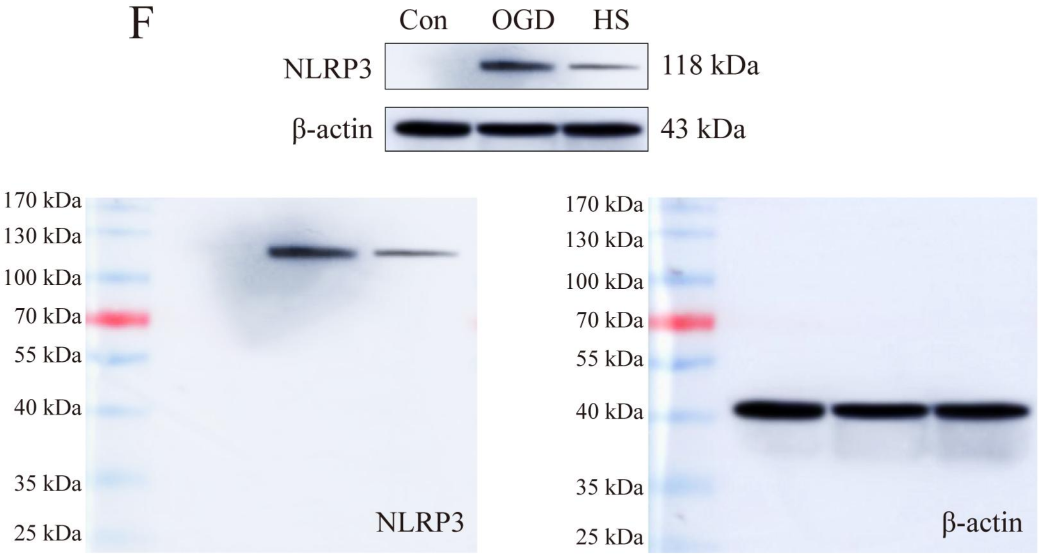
Con OGD HS
NLRP3
118 kDa
β-actin
43 kDa
170 kDa
130 kDa
100 kDa
70 kDa
55 kDa
40 kDa
35 kDa
25 kDa
NLRP3
β-actin

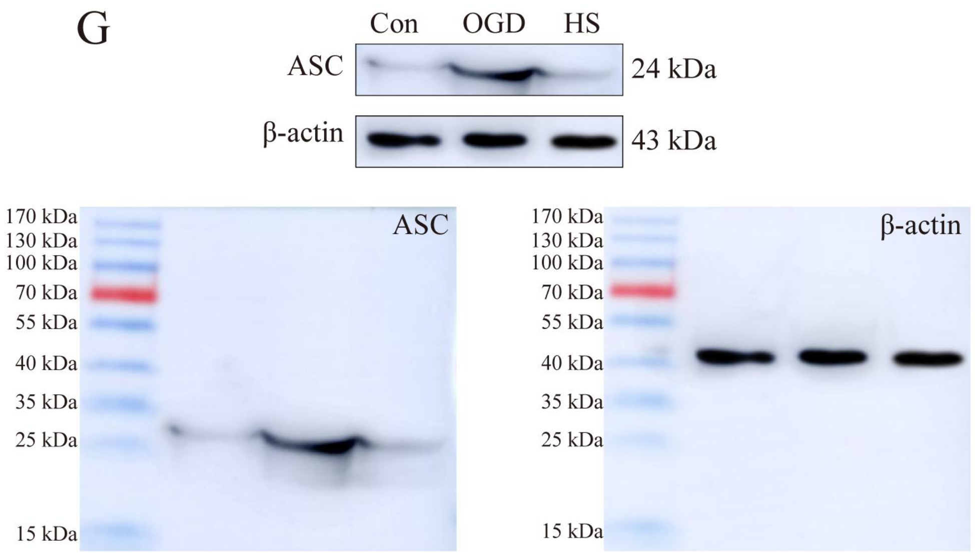
G
Con
OGD
HS
ASC
24 kDa
β-actin
43 kDa
170 kDa
130 kDa
100 kDa
70 kDa
55 kDa
40 kDa
35 kDa
25 kDa
15 kDa
ASC
β-actin

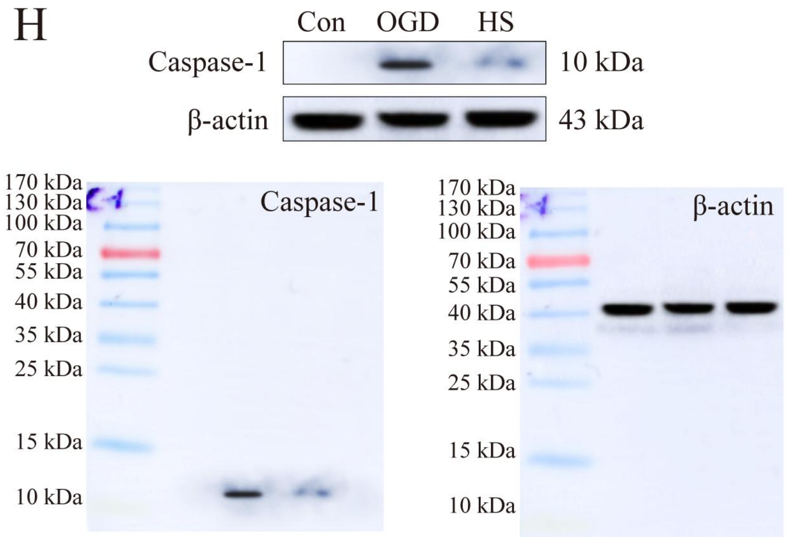
H
Con
OGD
HS
Caspase-1
10 kDa
β-actin
43 kDa
170 kDa
130 kDa
100 kDa
70 kDa
55 kDa
40 kDa
35 kDa
25 kDa
15 kDa
10 kDa
Caspase-1
β-actin

I

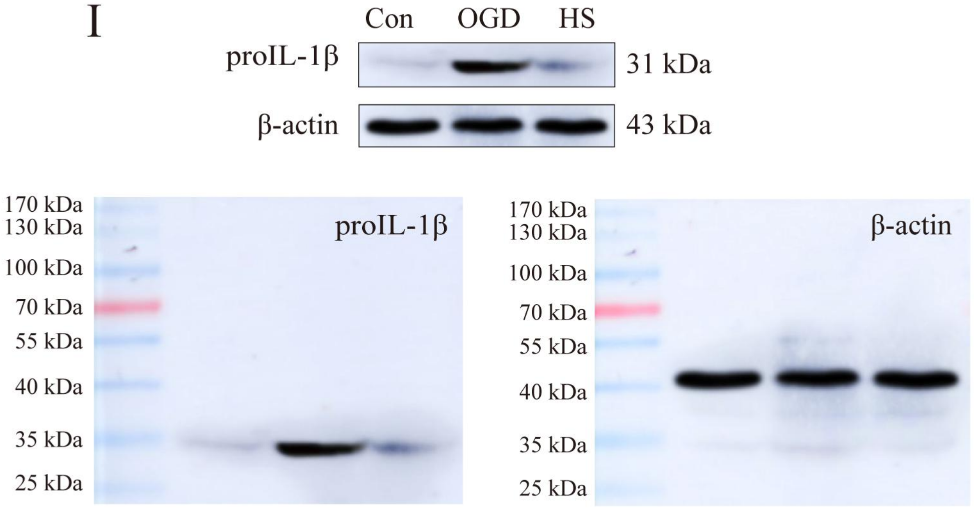
Con
OGD
HS
proIL-1β
31 kDa
β-actin
43 kDa
170 kDa
130 kDa
100 kDa
70 kDa
55 kDa
40 kDa
35 kDa
25 kDa
proIL-1β
β-actin

J

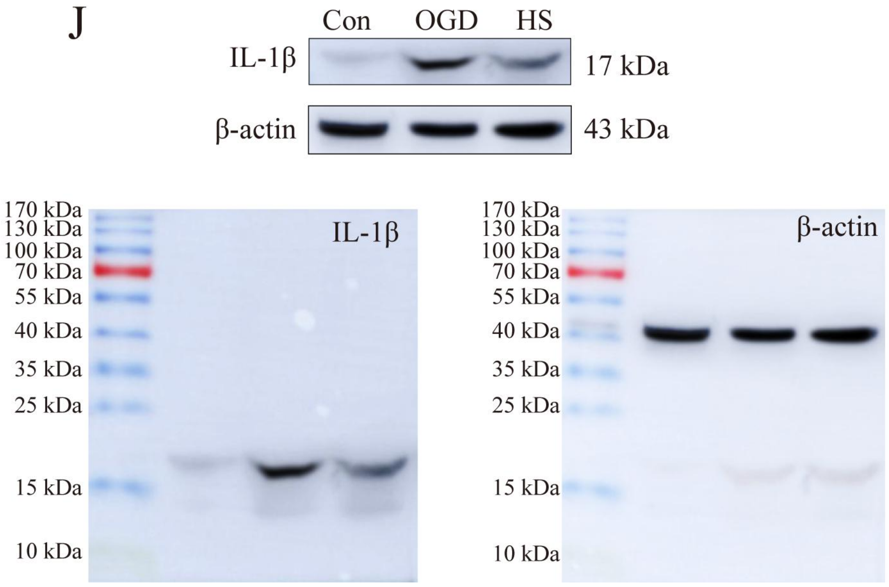
Con
OGD
HS
IL-1β
17 kDa
β-actin
43 kDa
170 kDa
130 kDa
100 kDa
70 kDa
55 kDa
40 kDa
35 kDa
25 kDa
15 kDa
10 kDa
IL-1β
β-actin

**Supplementary Figure 11** Full unedited blots for Figure 5.

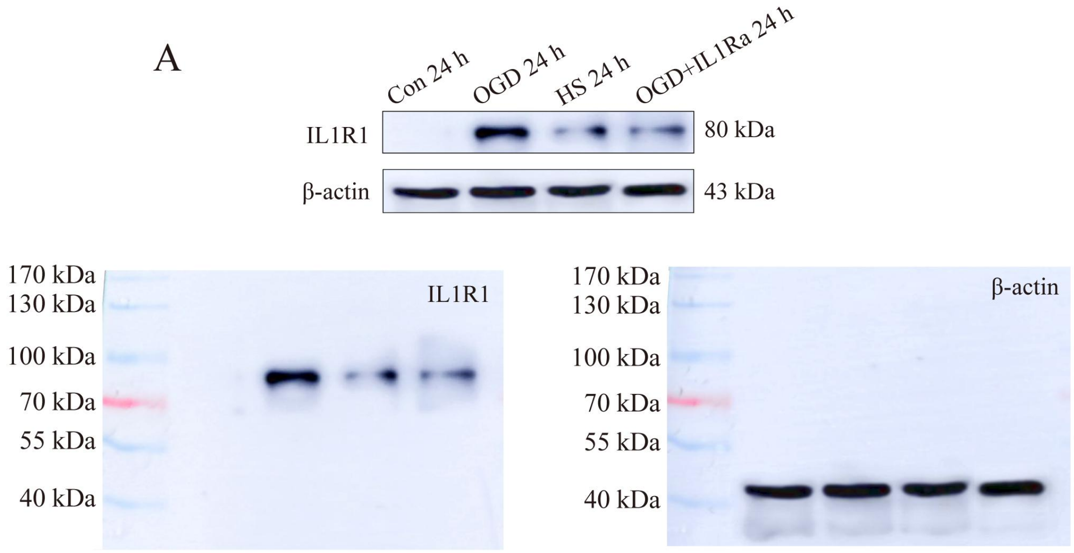


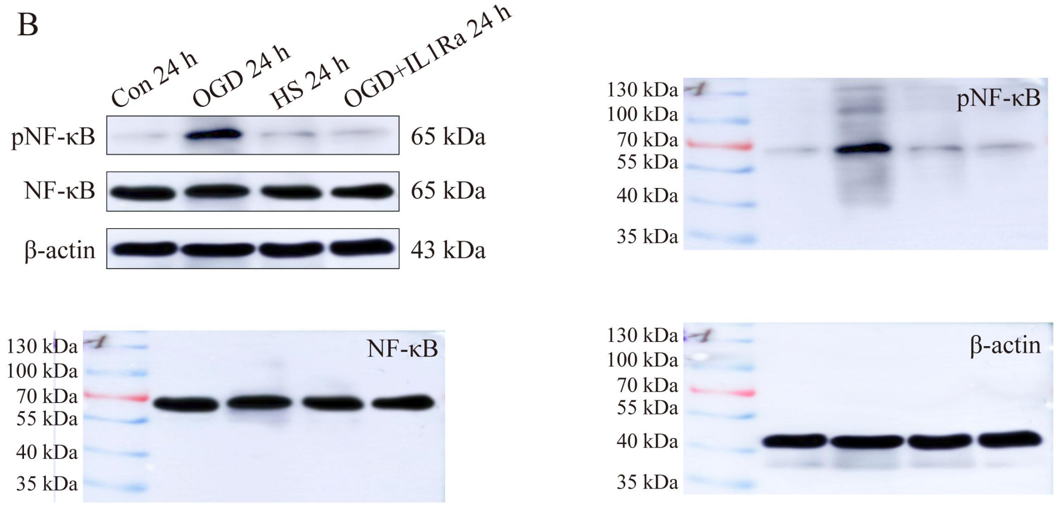

C

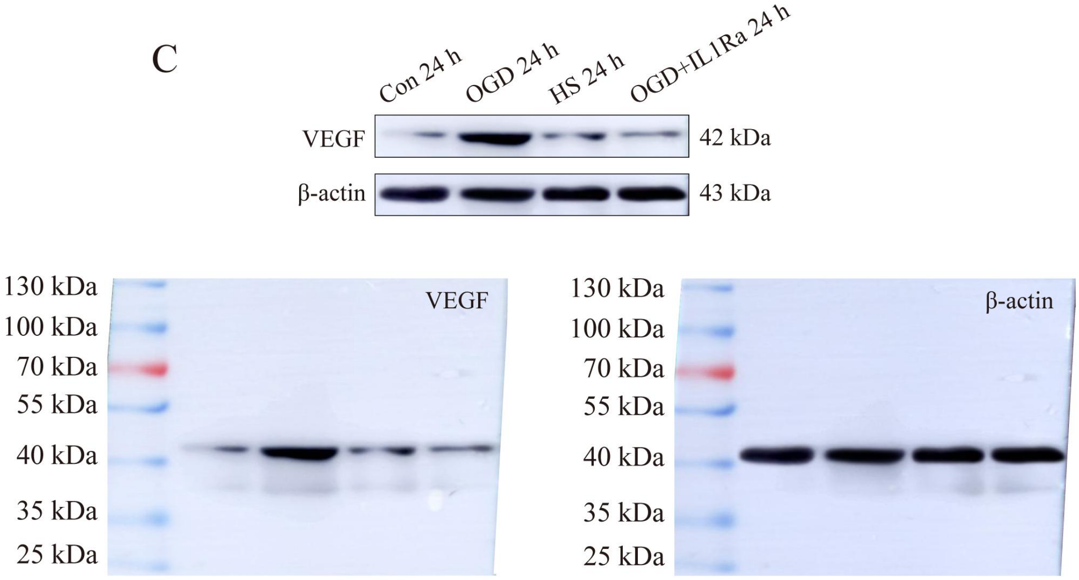
Con 24 h
OGD 24 h
HS 24 h
OGD+IL1Ra 24 h
VEGF
42 kDa
β-actin
43 kDa
130 kDa
100 kDa
70 kDa
55 kDa
40 kDa
35 kDa
25 kDa
VEGF
130 kDa
100 kDa
70 kDa
55 kDa
40 kDa
35 kDa
25 kDa
β-actin

D

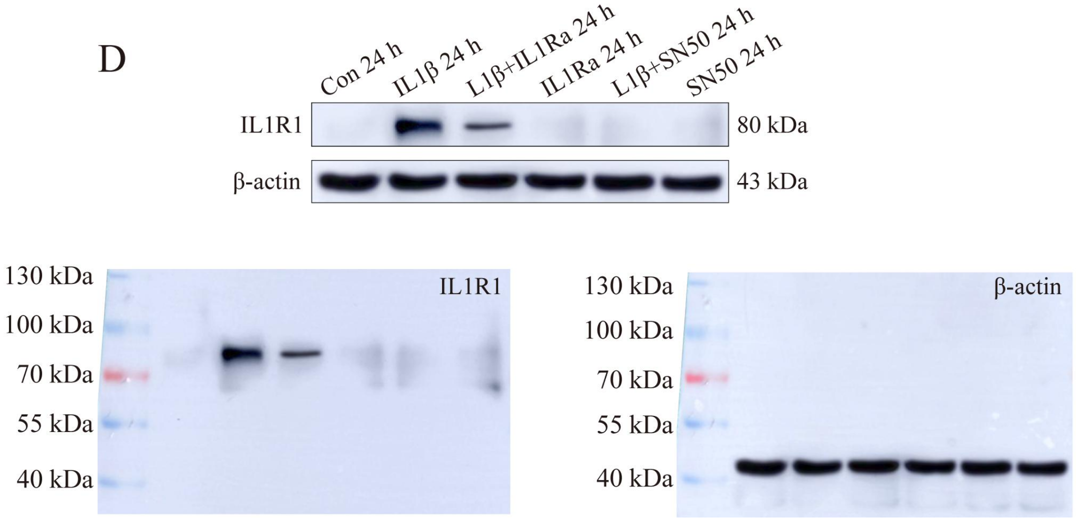
Con 24 h
IL1β 24 h
L1β+IL1Ra 24 h
IL1Ra 24 h
L1β+SN50 24 h
SN50 24 h
IL1R1
80 kDa
β-actin
43 kDa
130 kDa
100 kDa
70 kDa
55 kDa
40 kDa
IL1R1
130 kDa
100 kDa
70 kDa
55 kDa
40 kDa
β-actin

E

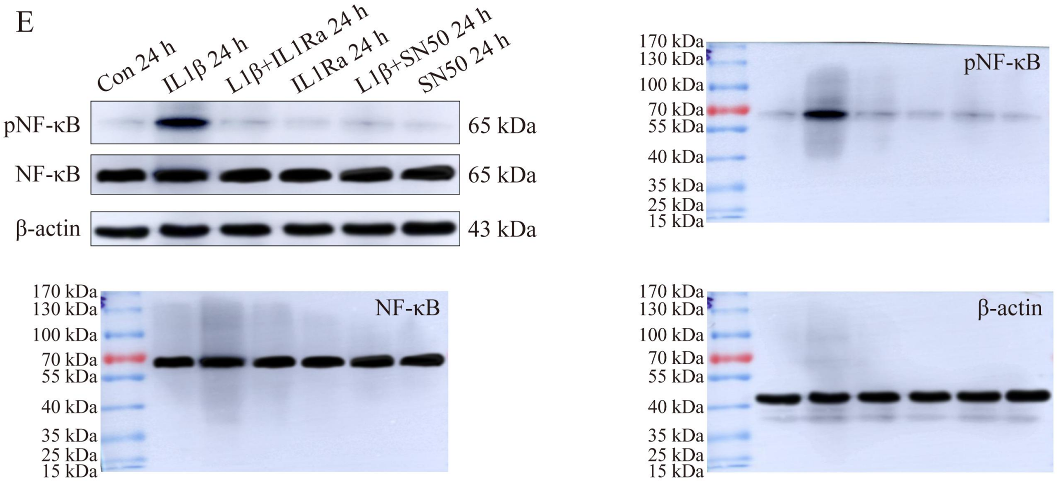

Con 24 h
IL1β 24 h
L1β+IL1Ra 24 h
IL1Ra 24 h
L1β+SN50 24 h
SN50 24 h
pNF-κB
65 kDa
NF-κB
65 kDa
β-actin
43 kDa
170 kDa
130 kDa
100 kDa
70 kDa
55 kDa
40 kDa
35 kDa
25 kDa
15 kDa
pNF-κB
NF-κB
β-actin


F

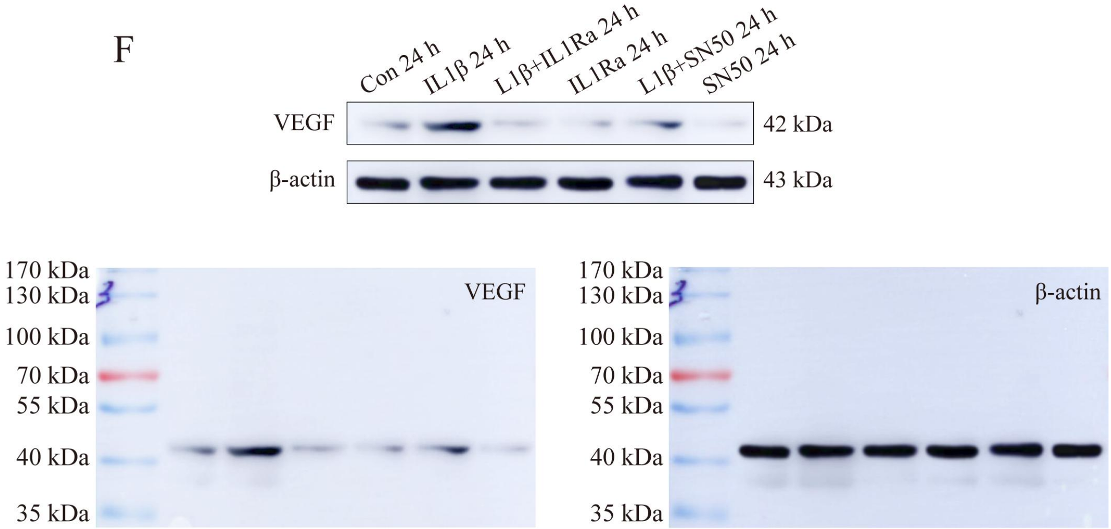

Con 24 h
IL1β 24 h
L1β+IL1Ra 24 h
IL1Ra 24 h
L1β+SN50 24 h
SN50 24 h
VEGF
42 kDa
β-actin
43 kDa
170 kDa
130 kDa
100 kDa
70 kDa
55 kDa
40 kDa
35 kDa
VEGF
β-actin

**Supplementary Figure 12** Full unedited blots for Supplementary Figure 6.

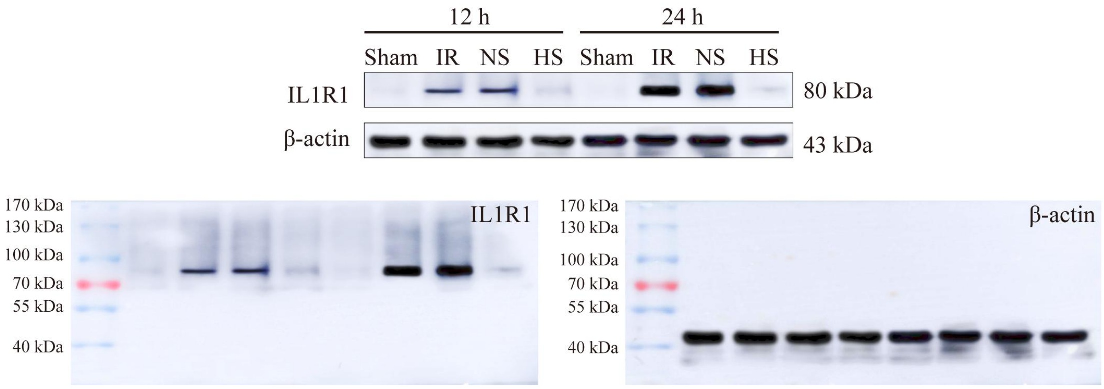


**Supplementary Figure 13** Full unedited blots for Supplementary Figure 7.

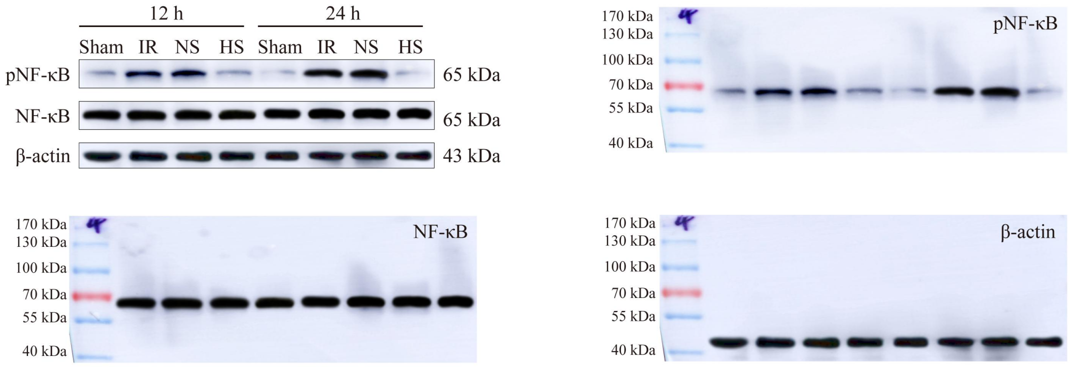

Supplement: Supplementary file 1 — Fig S1‐S13 [file CNS-26-1045-s001.pdf]
